# The RNA and protein landscapes of mouse brain organoids

**DOI:** 10.1016/j.isci.2026.118144

**Published:** 2026-11-05

**Authors:** Anne-Charlotte Fromaget, Céline Gonthier-Guéret, Khadija El Koulali, Sophie Périllous, Nuttamonpat Gumpangseth, Bertille Montibus, Philippe Arnaud, Serge Urbach, Franck Court, Tristan Bouschet

**Affiliations:** 1 IGF, University of Montpellier, CNRS, INSERM, Montpellier, France; 2 Genetics, Reproduction and Development Institute (iGReD), CNRS, INSERM, University Clermont Auvergne, 63000 Clermont-Ferrand, France; 3 BCM, University of Montpellier, CNRS, INSERM, Montpellier, France

**Keywords:** brain organoids, transcriptomics, proteomics, stem cells, alternative polyadenylation, alternative splicing, synaptic proteins

## Abstract

Organoids are powerful models of brain development and function. Mouse brain organoids reproduce the main steps of neurodevelopment *in vivo* and differentiate more rapidly than human organoids, making them scalable for screening applications. However, mouse brain organoids remain poorly defined. Here, we showed that the transcriptome and proteome of mouse brain organoids developed for three weeks are close to those of a neonatal mouse brain. Strikingly, organoids recapitulated the majority of alternative splicing and polyadenylation-site events that define neural identity *in vivo*. Proteomics revealed that most changes in mRNA expression were translated into proteins. Mouse brain organoids differentiated for only three weeks already harbored a complex network of synaptic proteins, including the ionotropic and metabotropic receptors for the neurotransmitters glutamate and GABA. To conclude, our study provides evidence that mouse brain organoids are a rapidly maturing and relevant model of mammalian brain development and function in a neonatal-like environment.

## Highlights

• In three weeks, mouse brain organoids acquire a neonatal-like transcriptome
• Brain organoids reasonably mimic *in vivo* alternative splicing and polyadenylation events
• The proteomic profiles of mouse brain organoids and the newborn brain are close
• Most mRNAs are translated into proteins

## Graphical abstract

**Figure:**
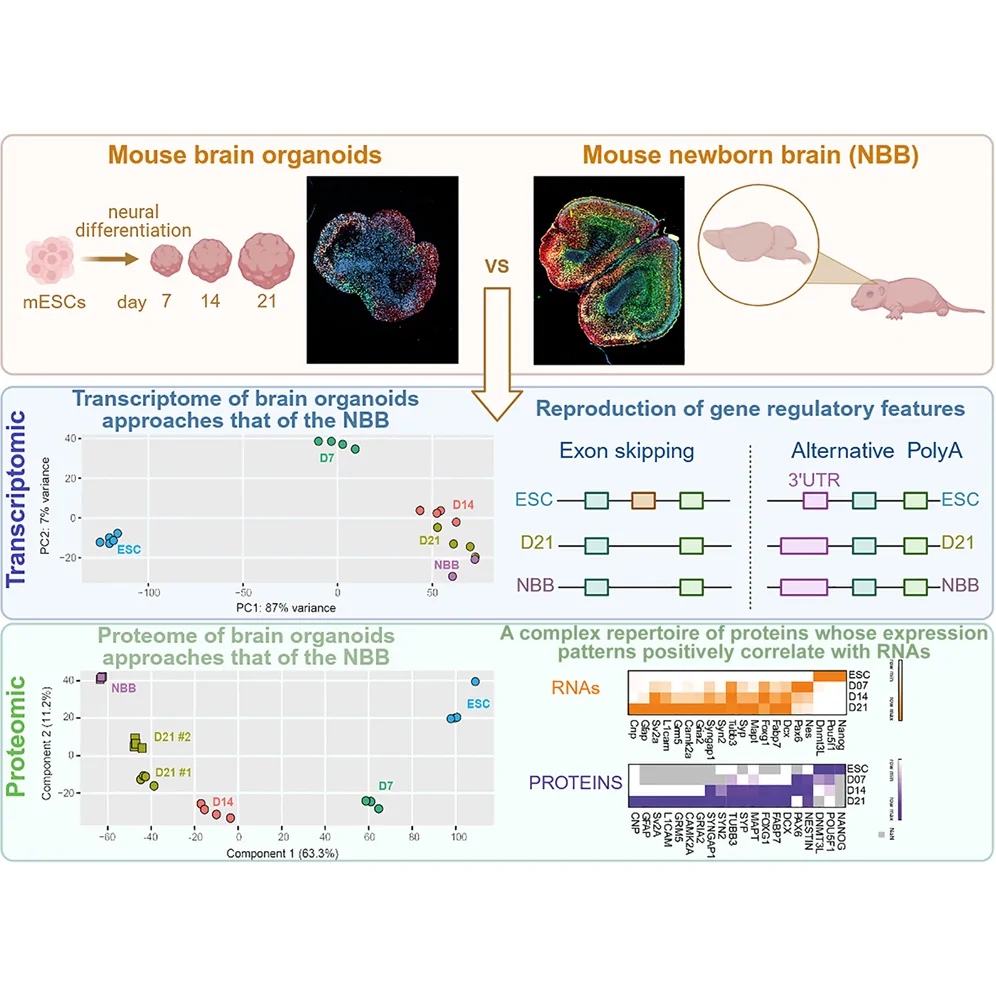

## Teaser

Neuroscience; Stem cells research; Developmental biology; Proteomics; Transcriptomics

## Introduction

Since the landmark study by Yoshiki Sasai’s team describing the spontaneous self-organization of dissociated mouse and human embryonic stem cells (ESCs) into brain-like tissues,^1^ brain organoids have emerged as a powerful tool for modeling brain development and function.

While human brain organoids capture specific features of human neurodevelopment, mouse brain organoids have several advantages over their human counterparts. First, brain organoids generated from mouse ESCs (mESCs) complete neuronogenesis in only three weeks, compared to several months for human organoids.^2^ Mouse brain organoids recapitulate the main steps of neurogenesis that take place in most mammals.^1^^,^^3^^,^^4^ The mouse is the predominant model for preclinical studies in the brain,^5^ and ESCs can be isolated from mouse lines.^6^^,^^7^ Lastly, even if a study comparing their reproducibility is lacking, the emerging picture is that the generation of mouse brain organoids is more reproducible than human organoids, possibly because the building plan of the mouse is simpler and therefore easier to reproduce *in vitro*, and the limited size of mouse organoids facilitates oxygen and nutrient diffusion, thereby limiting necrosis.

Similar to human pluripotent cells, mESCs can be specified into organoids of defined brain identities, including the cerebral cortex and the cortical hem,^1^^,^^3^ the striatum,^8^ and the hypothalamus.^9^ Mouse brain organoids recapitulate the cell diversity of the mouse brain, and they contain neural progenitors, neurons, and glial cells,^1^^,^^3^^,^^4^ in the expected proportions.^4^ Mouse brain organoids display spontaneous calcium waves^1^ and electrical activities resembling those recorded in embryonic brain slices,^3^ which indicates a certain degree of functional maturity.

In contrast to human brain organoids, whose transcriptome^10^^,^^11^ and proteome^12^^,^^13^ are well defined, the transcriptome of mouse brain organoids was only partly defined by single-cell RNA-seq,^4^ and their proteome is unknown.

Here, we have generated the transcriptome of mouse brain organoids at three developmental stages and showed that 21-day-old brain organoids resembled the mouse newborn brain. Beyond cell identity, we analyzed their transcriptome to an unprecedented level and asked whether brain organoids recapitulate two key gene regulatory features critical for neurodevelopment: exon skipping^14^ and alternative polyadenylation site usage.^15^ Finally, mESCs, brain organoids and mouse newborn brain were profiled by proteomics to shed light on the relative abundance of proteins, including synaptic proteins, and to estimate whether changes in RNA levels are effectively translated into proteins.

## Results

### Generation of brain organoids from mouse embryonic stem cells

mESCs were differentiated into brain organoids using a protocol^16^ inspired by the original protocol developed by Sasai and co-workers,^1^ which consists of aggregating mESCs into ultra-low adhesion wells in a medium supplemented with 10% Knock-out serum replacement (KSR), the BMP/Smad inhibitor DMH1 to promote neural induction, and the Wnt inhibitor IWP-2 to rostralize the neural tube (Figure 1A). After 7 days of culture, neural organoids were transferred to ultra-low adhesion 6-well plates in a maturation medium containing N2 and B27 without vitamin A,^16^ under orbital shaking to improve the transfer of nutrients and oxygen. The quality of differentiation was checked by immunofluorescence experiments performed on starting mESC cultures (Figure 1B) and cryosections of organoids developed for 7, 14, and 21 days (Figure 1C). As expected, this protocol led to a massive expansion in organoid size and the generation of neural progenitors (NESTIN and PAX6^+^ cells), neurons (ELAVL3^+^, TBR1^+^, TUBB3^+^ cells), and astrocytes (GFAP^+^ cells) (Figure 1C). These immunofluorescence data are consistent with our previous RT-qPCR experiments, which showed the expected expression pattern of cell fate markers in this model.^17^ As reported,^1^^,^^18^^,^^19^ mESCs differentiated into brain cells for three weeks share analogies with the newborn brain (NBB), including the expression of cortical layer markers TBR1 and BCL11B (also known as CTIP2) (Figures 1D, 1E, S1A and S1B).

**Figure 1:**
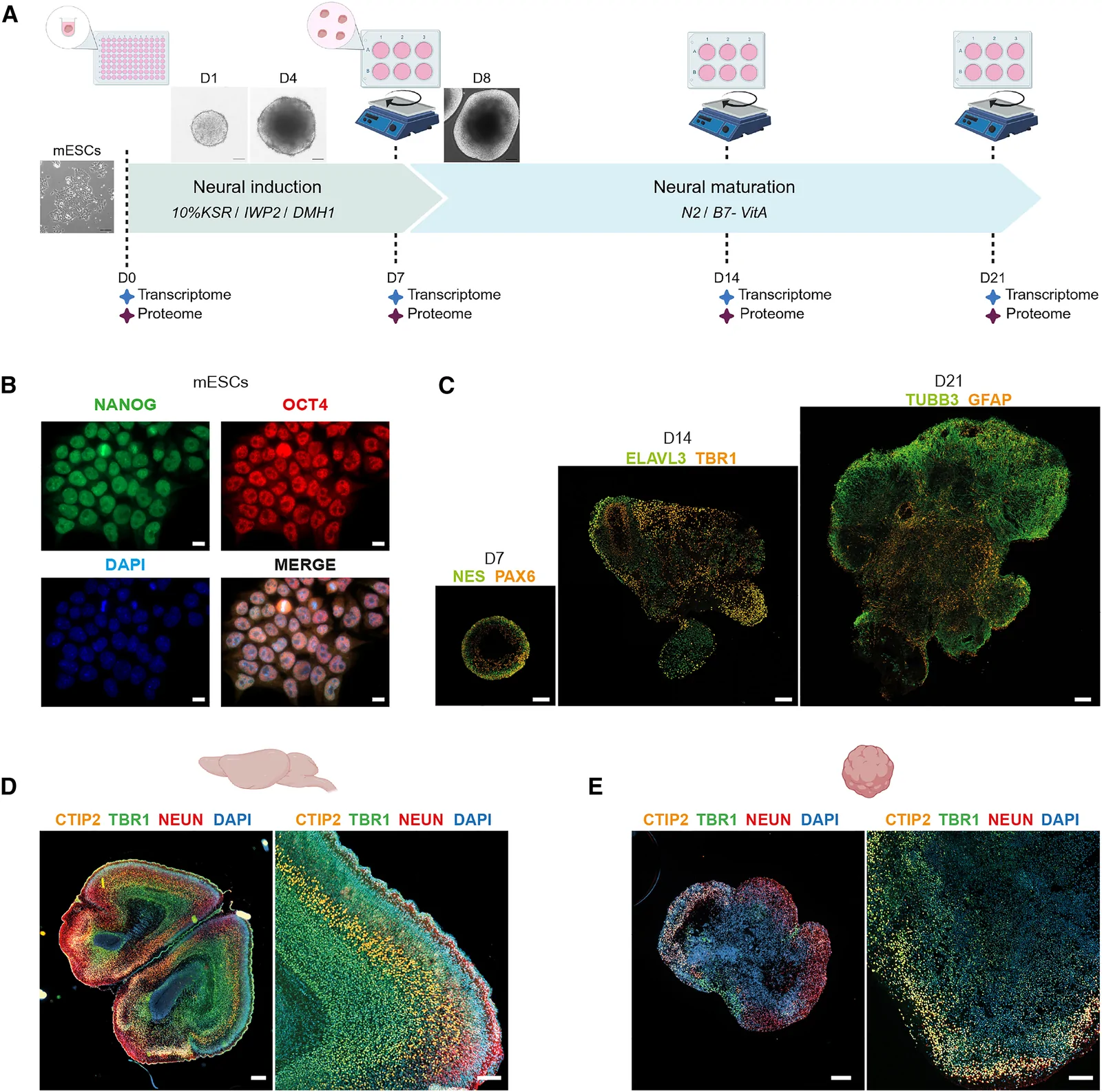
Brain organoid generation from mouse ESCs (A) Schematic timeline of brain organoid generation from mouse ESCs. mESCs and brain organoids developed for 7, 14, and 21 days were profiled by transcriptomics and proteomics. Part of the figure was created with BioRender. (B) Immunostaining for the pluripotency proteins NANOG (green) and POU5F1/OCT4 (red) in undifferentiated mESC cultures. The nuclei were stained with DAPI (blue). Scale bars, 10 μm. (C) Organoid cryosections were stained with NESTIN and PAX6 (at day 7), ELAVL3 and TBR1 (at day 14), and TUBB3 and GFAP (at day 21). Scale bars, 100 μm. (D) Left: expression of the cortical layer markers CTIP2 (orange) and TBR1 (green), co-stained with the neuronal marker NEUN (red) and DAPI (blue) in the NBB (at P1). Scale bars, 500 μm. Right: higher-magnification picture centered on the cerebral cortex. Scale bars, 100 μm. Part of the figure was created with BioRender. An extended version is shown in Figure S1. (E) Same immunostaining as in (D) but on a brain organoid section (at day 21). Scale bars, 200 μm (left), 100 μm (right). An extended version is shown in Figure S1.

### The transcriptome of mouse brain organoids progressively approached that of the neonatal mouse brain

Next, mESC-derived brain organoids (at days 7, 14, and 21) and NBB samples (used as a gold standard) were profiled by RNA-seq (Figure 2A). Principal component analysis (PCA) (Figure 2B) showed that mESCs clustered separately from brain organoids and the NBB samples. Organoids at day 7 were the closest to mESCs, then D14 organoids, and D21 organoids were the closest to NBB samples. This suggests that the transcriptome of brain organoids progressively approaches that of the NBB. Consistent with the PCA analysis, during the neural induction period (from mESCs to day 7), there was a sharp transition in gene expression, with 7988 differentially expressed genes (DEGs) (Figure S2A and Table S1). Then, during the neural maturation period (from day 7 onwards), the number of DEGs decreased to 2745 DEGs between D14 and D7 organoids, and 517 DEGs between D21 and D14 organoids (Figures S2A–S2C and Tables S2, S3, and S4). For neural induction, the significantly upregulated genes were enriched in Gene Ontology (GO)^20^ terms associated with neurodevelopment, including “nervous system development” (FDR: 1.1E−67) (Figure S2D) and “neurogenesis” (FDR: 3.02E−53), while downregulated genes were associated with “stem cell population maintenance” (FDR: 6.18E−08) (Table S5). This indicates that after 7 days of organoid development, most mESCs have exited pluripotency and adopted a neural fate. During the maturation period (from day 7 to day 21), the upregulated genes were enriched in terms associated with neuronal maturation (“regulation of presynaptic membrane potential”, FDR: 9.83E−29; “regulation of postsynaptic membrane potential”: 5.78E−27; “dendrite”: 4.25E−61; “glutamatergic synapse”: 6.36E−81), and downregulated genes with “cell cycle” (FDR: 2.03E−34) and “mitosis” (FDR: 4.42E−27) (Figure S2E and Table S6). Thus, GO enrichment analysis indicates that organoids rapidly acquire a neural identity and then progressively mature.

**Figure 2:**
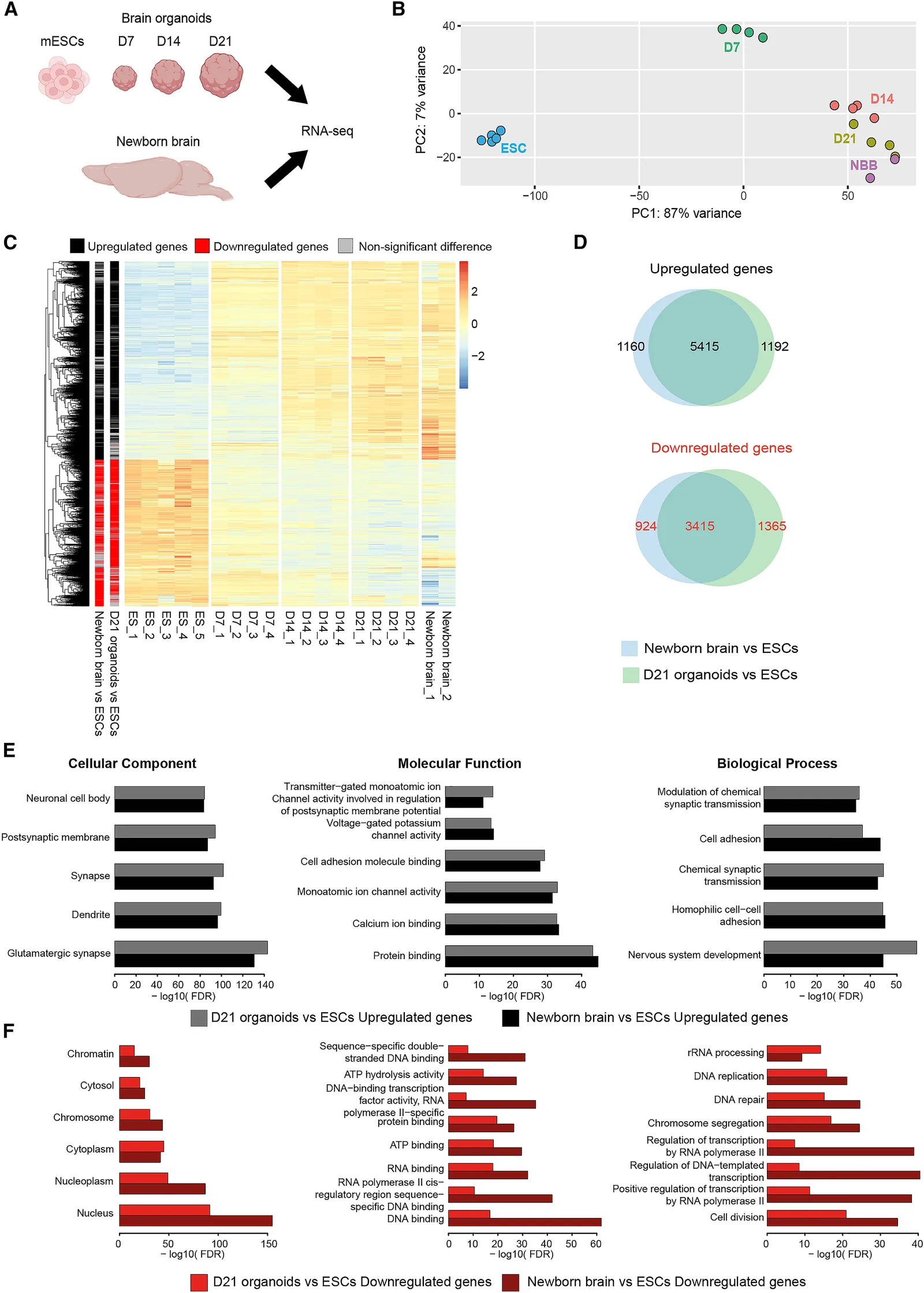
The transcriptome of mouse brain organoids progressively approaches that of the neonatal mouse brain (A) mESCs, brain organoids (at days 7, 14, and 21), and newborn brain (NBB) were profiled by RNA-seq. The figure was created with BioRender. (B) PCA shows the distribution of the transcriptomes of mESCs, brain organoids (at days 7, 14, and 21), and NBB samples. (C) Heatmap showing the centered-scaled expression levels of the differentially expressed genes between D21 organoids and the NBB, both compared to mESCs for all samples. Results of the differential analyses are shown on the left of the heatmap, with black for upregulated genes, red for downregulated genes, and gray for non-significant differences. (D) Venn diagrams show the overlapping upregulated and downregulated genes between D21 organoids and the NBB, both compared to mESCs. (E) Histograms of enriched GO terms in NBB vs. ESCs and D21 organoids vs. ESCs performed on upregulated genes (black and gray bars, respectively) determined using DAVID. (F) Histograms of enriched GO terms in NBB vs. ESCs and D21 organoids vs. ESCs performed on downregulated genes (red and light red bars, respectively).

Next, we compared the transcriptomic profiles of D21 brain organoids and the NBB, both compared to mESCs (because mESCs are derived from *in vivo* growing blastocysts,^6^^,^^7^ we reasoned they could serve as a reasonable starting developmental time point for both organoids and the NBB). Most of the DEGs between D21 organoids and mESCs (Table S7) and between the NBB and mESCs (Table S8) were shared: 5415/7767 (69.7%) upregulated genes and 3415/5704 (59.9%) downregulated genes were common (Figures 2C and 2D). Accordingly, brain organoids and NBB samples shared the same most significantly enriched GO terms (Tables S9 and S10). For upregulated genes (Figure 2E), this includes, “glutamatergic synapse” (FDR: 8,04E−144 for brain organoid, and 1.95e−131 for the NBB), “dendrite” (FDR: 2.56e−100 for brain organoid, and 4.48e−97 for the NBB), and “axon” (FDR: 9.79e−83 for brain organoid, and 8.68e−76 for the NBB) (Figure 2E and Tables S9 and S10). Among common terms between brain organoids and the native brain (all with an FDR < 1e−10), we also retrieved “transmitter-gated monoatomic ion channel activity involved in the regulation of postsynaptic membrane potential” and nervous system development” (Figure 2E). For downregulated genes (Figure 2F), the most significantly enriched terms were also common between organoids and the NBB, including “cell division”. Thus, GO analysis suggests that the transcriptome of D21 organoids is enriched in the same GO terms associated with brain function and development as the newborn brain.

### Brain organoids lacked the non-neural cell types present in the neonatal mouse brain

However, PCA, the heatmap reporting gene expression across samples, and Venn diagrams (Figures 2B–2D) indicated a partial difference between the transcriptome profiles of D21 brain organoids and NBBs. Next, we directly compared the transcriptomes of the NBB and D21 organoids. We identified 942 upregulated genes and 415 downregulated genes between the NBB and D21 organoids (Figures S3A and S3B and Table S11). The genes upregulated in the NBB were enriched in GO terms for “angiogenesis” (FDR:3.83E−09), “inflammatory response” (FDR: 4.26E−13), and “extracellular matrix” (1.08E−08) (Figure S3C and Table S12). This likely reflects that, compared to the native brain, organoids lack blood cells, a circulatory system, and cells triggering an inflammatory response in the brain (principally, microglia), all of which might participate in depositing a brain-specific matrix.

### Brain organoids largely reproduced alternative splicing events

Next, to extend our analysis of the transcriptomes of brain organoids and the NBB beyond cell identity, we asked whether gene regulatory features important for brain development are also well reproduced by organoids. We first focused on alternative splicing, which is tightly regulated during development and results in tissue-specific transcript isoforms.^14^ However, it remains unknown to what extent brain organoids (including human organoids) recapitulate the variety of transcript isoforms found in the *in vivo* brain. To compare splicing events occurring in the NBB and D21 organoids (both compared to mESCs), we employed the widely used statistical suite R-MATS -Robust and flexible detection of differential alternative splicing from replicate RNA-Seq data (Figure 3A).^21^ During differentiation, we observed a profound remodeling of transcripts both *in vivo* and *in vitro* (Figure 3B and Tables S13 and S14). Some genes were affected by several alternative splicing events (hence, the number of genes < the number of events). Most of the changes in transcript isoforms were related to exon skipping, which results in either exon inclusion or exclusion (Figure 3B). Thus, we next focused on comparing the *in vivo* and *in vitro* situations for exon skipping. Strikingly, of the 1,254 genes with skipped exons in the newborn brain, 1,002/1,254 (80%) were also present in D21 brain organoids (Figure 3C). Interestingly, the number of skipped exon events increased during organoid development (Figure S4A), and the correlation between the R-MATS metric of the NBB and organoids also increased as development progressed (Figure S4B). Thus, collectively, this suggests that brain organoids progressively acquire part of the repertoire of the splice isoforms of the NBB.

**Figure 3:**
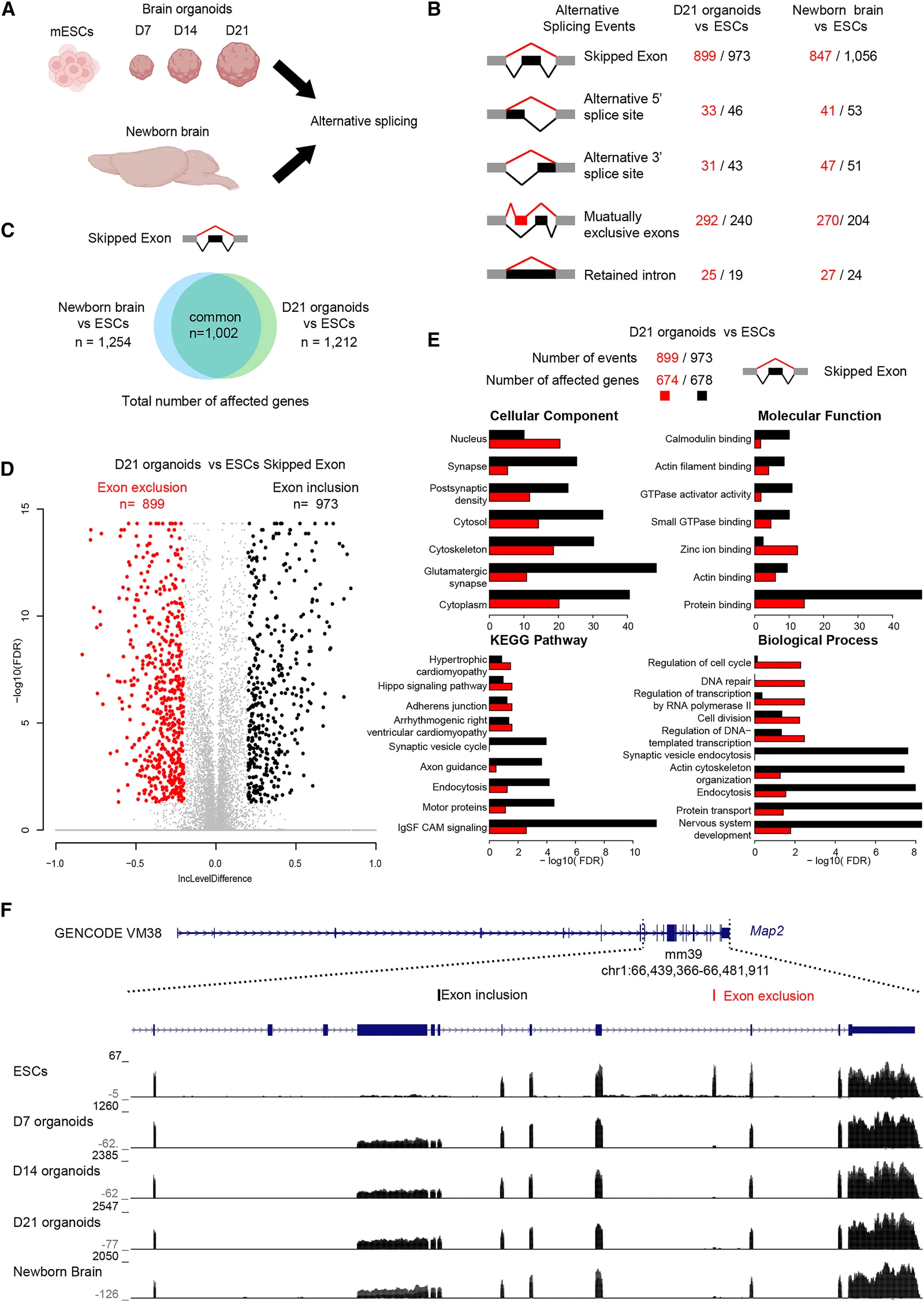
Brain organoids largely reproduce exon skipping events (A) Comparison of alternative splicing events in brain organoids and the newborn brain. (B) Alternative splicing events detected using R-MATS in the NBB and D21 organoids compared to mESCs. Red and black numbers correspond to the nature of the alternative splicing events (red: exon exclusion; black: exon inclusion). (C) Venn diagram shows the number of overlapping genes affected by skipped exon events between brain organoids at day 21 and the NBB, both compared to mESCs. (D) Volcano plot of exon skipping events in D21 organoids compared to mESCs. There were 899 events of exon exclusion (red) and 973 events of exon inclusion (black). Alternative splicing events were identified when |IncLevelDifference| > 0.2 and FDR < 0.05. (E) Histogram of GO analysis (KEGG pathway, cellular component, molecular function, and biological process) of genes affected by exon exclusion (red) or inclusion (black) in D21 organoids compared to mESCs. (F) UCSC genome browser capture of RNA-seq data of mESCs, brain organoids (D7, D14, and D21), and NBB for reads mapping to the *Map2* gene. The genomic location of the excluded exon is indicated by vertical red bars, and inclusion by black bars.

Next, we performed a GO analysis on the genes displaying either exon inclusion (973 genes) or exclusion (899 genes) in D21 organoids compared to mESCs (Figure 3D and Tables S13, S14, and S15). GO analysis revealed an enrichment in neural terms, including “synaptic vesicle cycle”, “axon guidance”, “synaptic vesicle endocytosis”, “nervous system development”, and “glutamatergic synapse”, and this was independent of the nature of events (exon inclusion or exclusion) (Figure 3E). Among the list of genes that were common between the NBB and D21 organoids, we noticed the presence of *Map2*, a gene involved in neurite outgrowth, synaptic plasticity, and transport.^22^ A screen capture of RNA-seq data uploaded into the UCSC Genome Browser^23^ confirmed that, compared to the ESC stage, *Map2* experienced both exon inclusion and exon exclusion, leading to similar *Map2* transcript isoforms in the organoids and the newborn brain (Figure 3F). Thus, collectively, these data suggest that alternative splicing events, notably exon skipping, are well reproduced by brain organoids, and they are associated with a neuronal signature.

### Brain organoids largely reproduced alternative polyadenylation events

Another gene regulatory feature important for neurodevelopment is alternative polyadenylation (APA), which results in either 3′UTR lengthening or shortening.^15^ APA is context-dependent and important for transcript location, stability, and translation.^15^ In the developing brain, a defined subset of transcripts is targeted by APA, which most often results in 3′UTR lengthening.^24^^,^^25^ However, it remains elusive whether brain organoids recapitulate the APA site usage of the *in vivo* brain. Thus, we next sought to identify genes experiencing APA in brain organoids and compare that list to the *in vivo* brain (Figure 4A). To this aim, we used the bioinformatics package APAlyzer.^26^ When compared to the ESC stage, we identified 254 genes with APA in organoids at day 7 (Figure S5A and Table S16), 331 genes at day 14 (Figure S5B and Table S17), 380 genes in organoids at day 21 (Figure 4B and Table S18), and 323 genes for the NBB (Figure 4C and Table S19). For all samples, the majority of APA resulted in 3′UTR lengthening (Figures 4B, 4C, S4A and S4B), as previously reported for the mouse brain.^24^^,^^25^ More specifically, in brain organoids at day 21, 337 transcripts lengthened while 43 shortened. For the NBB, 295 transcripts extended and 28 shortened (Figures 4B and 4C). Remarkably, 162/295 (55%) transcripts that extended in the NBB also extended in D21 brain organoids (Figure 4D). An APA usage heatmap confirmed that D21 organoids globally mimic the NBB situation (Figure 4E). Among the genes identified by APAlyzer, we noticed the presence of the RNA-binding protein *Elavl1*, known to be longer in the brain^27^, and the scaffolding protein *Homer2*. The extension of the 3′UTR of *Elavl1* and *Homer2* was confirmed by examination of RNA-seq data (Figures 4F and 4G). The 337 genes whose transcripts lengthen in D21 organoids were enriched in GO terms related to the ubiquitin-proteasome pathway, including “Ubl conjugation” (FDR: 9.81E−11) and “Ubiquitin mediated proteolysis” (FDR: 0.000036) (Figure S5C and Table S20). Thus, brain organoids recapitulated most of the APA events taking place *in vivo*. This resulted in 3′UTR lengthening over shortening and an enrichment in genes related to the ubiquitin-proteasome pathway.

**Figure 4:**
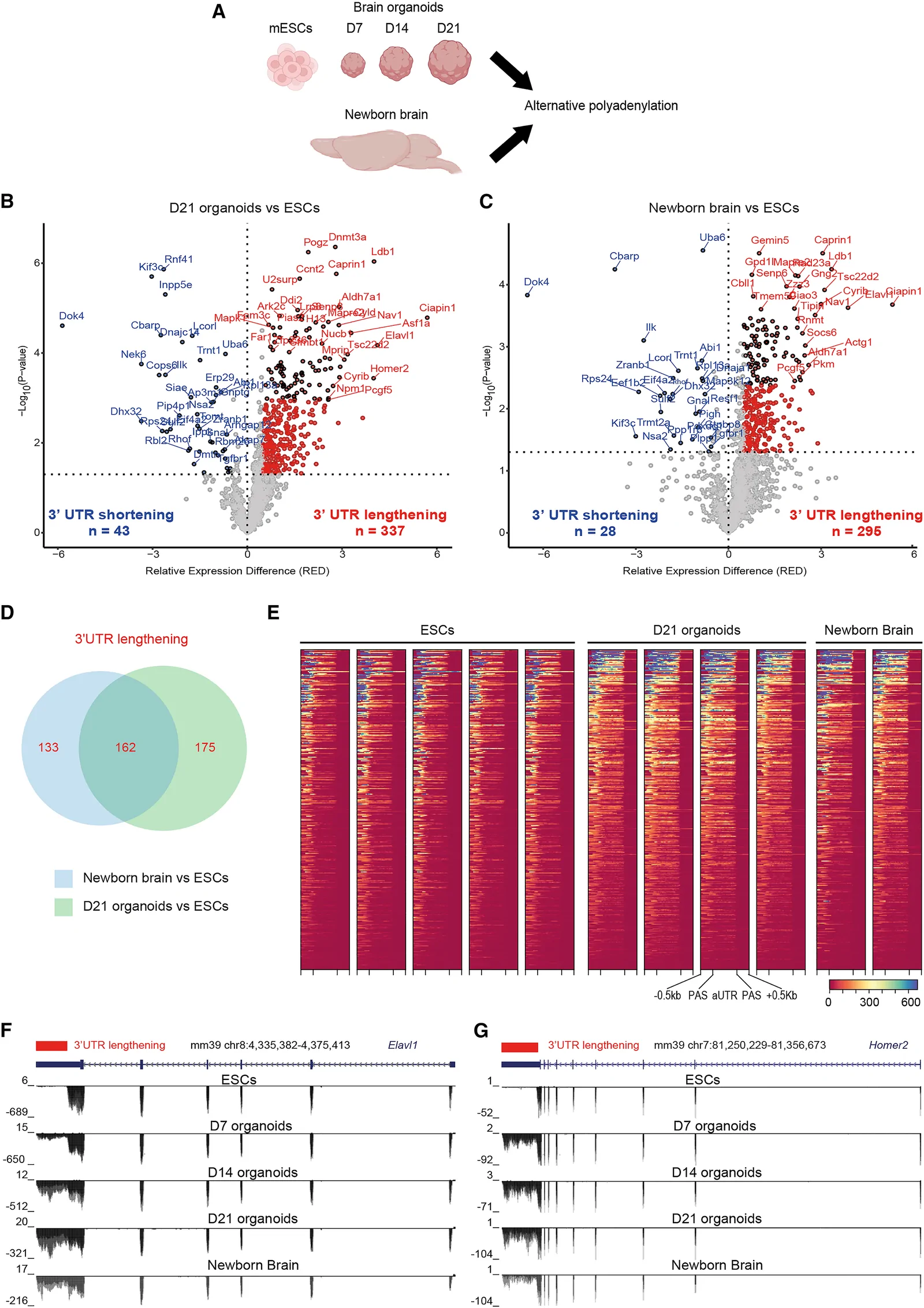
Brain organoids largely reproduce alternative polyadenylation site usages (A) Comparison of alternative polyadenylation site usage in brain organoids and the newborn brain. (B) Volcano plot of the distribution of genes identified with alternative polyadenylation site usage in D21 organoids compared to mESCs, indicating whether a proximal (blue, resulting in 3′UTR shortening) or a distal (red, resulting in 5′UTR shortening) site was used. Genes were identified using APAlyser with the following cut-offs: |RED| (Relative Expression Difference) between proximal and distal polyadenylation site usage >0.5 and *p* value < 0.05. (C) Same as in B, but for the NBB compared to mESCs. (D) Venn diagram shows the number of overlapping genes displaying 3′UTR lengthening in NBB and D21 organoids. (E) APA usage heatmaps in ESCs, D21 organoids, and NBB samples for genes identified by comparing D21 organoids to ESCs. (F) UCSC genome browser capture of RNA-seq data of mESC, brain organoids (D7, D14, and D21), and NBB for reads mapping to the *Elavl1* gene. The genomic location of the site with APA usage is indicated by horizontal red bars. (G) Same as in F, but for the *Homer2* gene.

### The proteome of developing brain organoids encompasses morphogens, chemoattractive, and repulsive cues, cortical-layer markers, and synaptic proteins

While RNA-seq is widely used on human brain organoids,^10^^,^^11^ proteomics studies are still rare,^12^^,^^13^ and only one study compares the transcriptome and proteome of human brain organoids.^28^ In addition, the proteome of mouse brain organoids is unknown. Thus, we next sought to characterize the proteome signature of mouse brain organoids at days 7, 14, and 21 and compare them to their transcriptome. To this aim, mESC (*n* = 4 samples), and brain organoids at day 7 (*n* = 3), day 14 (*n* = 4), and day 21 (*n* = 4) were analyzed by Liquid Chromatography coupled to tandem Mass Spectrometry -LC-MSMS-, performed on an Orbitrap device (see *Methods*) (Figure 5A and Table S21). This approach yielded the identification of 5901 proteins with a cut-off of >3 peptides per protein (Table S21). In PCA, the ESC and D21 organoid samples clustered apart, with D7 and D14 organoids in between them (Figure 5B), as observed for RNA-seq (Figure 2B). Accordingly, a heatmap representing variation in protein expression across samples revealed massive changes in protein expression during the differentiation process, in particular during neural induction (Figure 5C). Next, we identified differentially expressed proteins (logFC > 2, and *p* > 0.01) during the transition from mESC to D7 organoids (Figure 5D), from D7 to D14 organoids (Figure 5E), and from D14 to D21 organoids (Figure 5F). As suggested by the distance between the four developmental stages in the PCA, more proteins were differentially expressed between ESC and day 7 (404 proteins) and between day 7 and day 14 (527 proteins) than between D14 and D21 (74 proteins). This indicates that there are sharp transitions in the proteome signature from ESC to day 7 and from D7 to D14, while the proteome is relatively stable between D14 and D21. As expected, we confirmed the selective expression of proteins that mark cell identity: the stemness marker NANOG was enriched in mESCs and the neural progenitor marker NESTIN in D7 organoids (Figure 5D). The radial glia marker FAPBP7 and the axonal marker MAPT were enriched at day 14 (compared to day 7) (Figure 5E). D21 organoids were enriched (compared to D14 organoids) in the mature neuronal markers THY1, GRM5, and CAMK2B, in the oligodendrocyte marker CNP, and in the astrocyte marker GFAP (Figure 5F).

**Figure 5:**
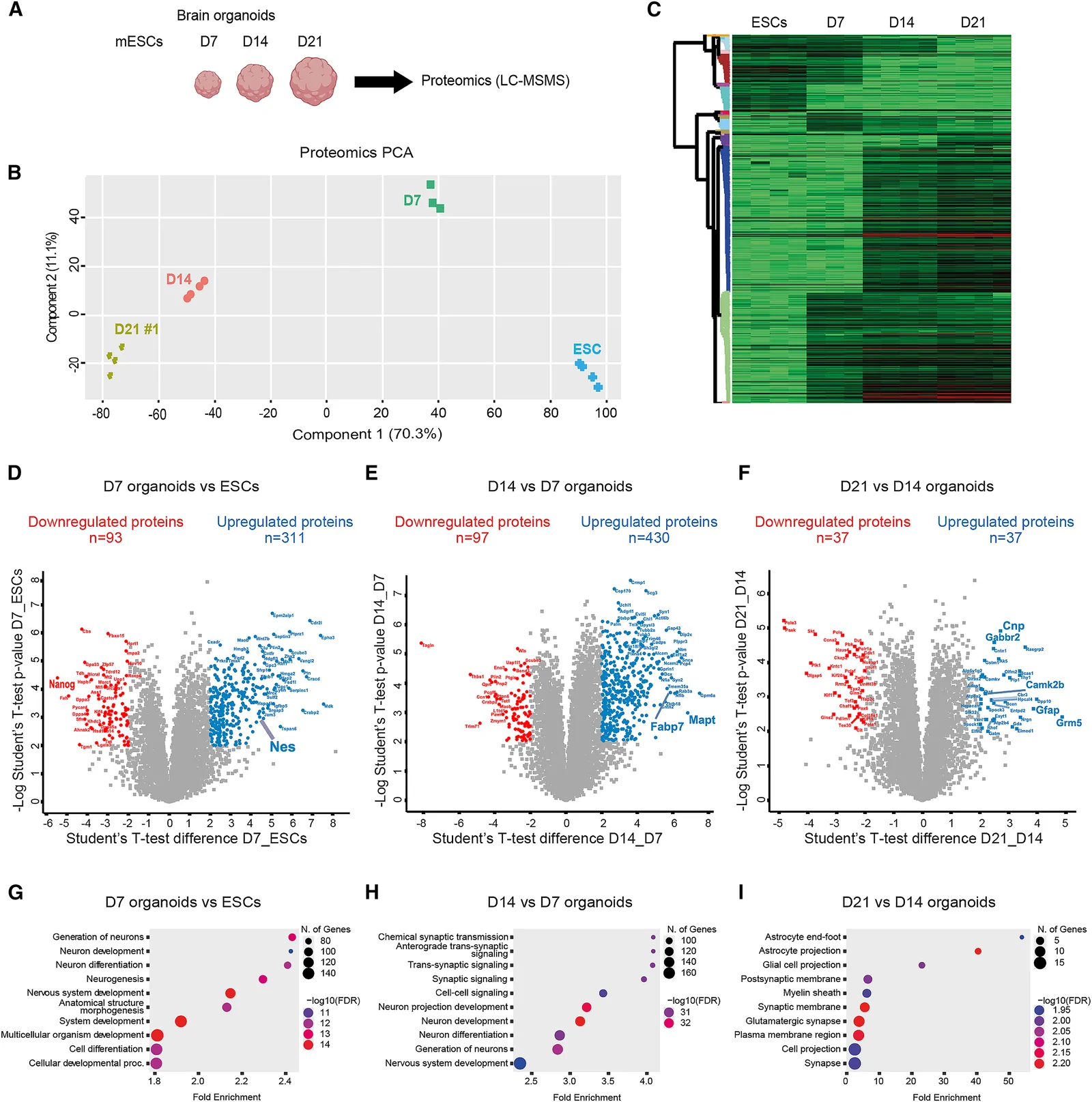
The proteome of developing mouse brain organoids (A) mESCs and brain organoids (at days 7, 14 and 21) were profiled by proteomics. LC-MS/MS: liquid chromatography coupled to tandem Mass Spectrometry. (B) PCA shows the distribution of the proteomes of mESCs and brain organoids (at days 7, 14, and 21). (C) Hierarchical clustering based on protein abundance (distance = Pearson) of proteins differentially expressed in at least one comparison (D7 vs. ESC, D14 vs. D7, D7 vs. D21). (D–F) Volcano plots of differentially expressed proteins between D7 organoids and ESC (C), D14 and D7 organoids (D), and D21 and D14 organoids (E). Proteins significantly downregulated are in red, and upregulated proteins are in blue. A selection of cell fate markers is written in a larger font. (G–I) Histograms of GO terms determined using ShinyGO on upregulated proteins between D7 organoids and mESCs (G), D14 and D7 organoids (H), and between D21 and D14 organoids (I).

Next, to infer the identity of organoids via their proteome, we performed a gene set enrichment analysis on differentially expressed proteins (DEPs) across the three developmental transitions. Gene set enrichment analysis was performed using ShinyGO.^29^ ShinyGO is a graphical enrichment tool where the query list of genes or proteins is interrogated toward a series of databases, including the Gene Ontology (GO) Consortium, the KEGG (Kyoto Encyclopedia of Genes and Genomes) for biological pathways, and the STRING database for Protein-protein interaction networks.^29^ In organoids at day 7 (compared to mESCs), the top enriched pathways included “multicellular organism development” (FDR: 6.9E−15), “central nervous system development” (FDR: 4.0E−09), and “neuron differentiation” (FDR: 8.4E−13) (Figure 5G). Strikingly, the proteins enriched in D14 organoids compared to D7 organoids indicated an additional sharp transition toward maturity with terms such as “chemical synaptic transmission” (FDR: 1.1E−31) and “neuron projection development” (FDR: 4.0E−33) (Figure 5H). In D21 organoids compared to D14, there was no enrichment in biological process terms (not shown). However, proteins were enriched for cellular component terms, suggesting that organoids continued maturing (“Glutamatergic synapse”, FDR: 6.0E−03; “Myelin sheath”: 1.1E−02, and “astrocyte endfoot”, 1.1E−02) (Figure 5I). Thus, these experiments suggest that the proteome drastically evolved between mESC and D7 organoids and between D7 and D14 organoids, and then slightly evolved.

Morphogens shape the developing brain.^30^^,^^31^ Brain organoids lack the organizers that secrete morphogens *in vivo*; accordingly, the identity of organoids is triggered by mimicking the influence of organizers through the addition of exogenous morphogens^32^ or by including morphogen centers.^33^ Of interest, our organoid model mimics the development of the most anterior part of the neural tube, known to develop in the absence of morphogens.^1^ However, we cannot rule out that our organoids generated “by default” produce their own morphogens. The identity of secreted morphogens by organoids is elusive. Thus, we next sought to determine the repertoire of morphogens, growth factors and cognate receptors intrinsically expressed by brain organoids. As shown in Figure 6A, we detected WNT7B and WNT8B proteins (together with the WNT receptors FZD2 and FZD7), which all peaked at day 7. BMP1 and BMPR2 were detected at later time points, consistent with a role in astrocyte generation and maintenance.^34^ We did not detect SHH, any FGFs, R-SPONDINs, or their receptors (SMO, FGFRs, and LGR5, respectively). The EGFR, important for the fate of neural stem cells (NSCs),^35^ was also expressed, but not the EGF. The EGFR is likely functional since we could expand single cells dissociated from organoids in the presence of EGF (and FGF2) (Figures S6A and S6B). These cells had typical NSCs’ properties (self-renewal, expression of the radial glia marker FABP7, and the ability to generate neurons and astrocytes upon removal of growth factors) (Figures S6C–S6E).

**Figure 6:**
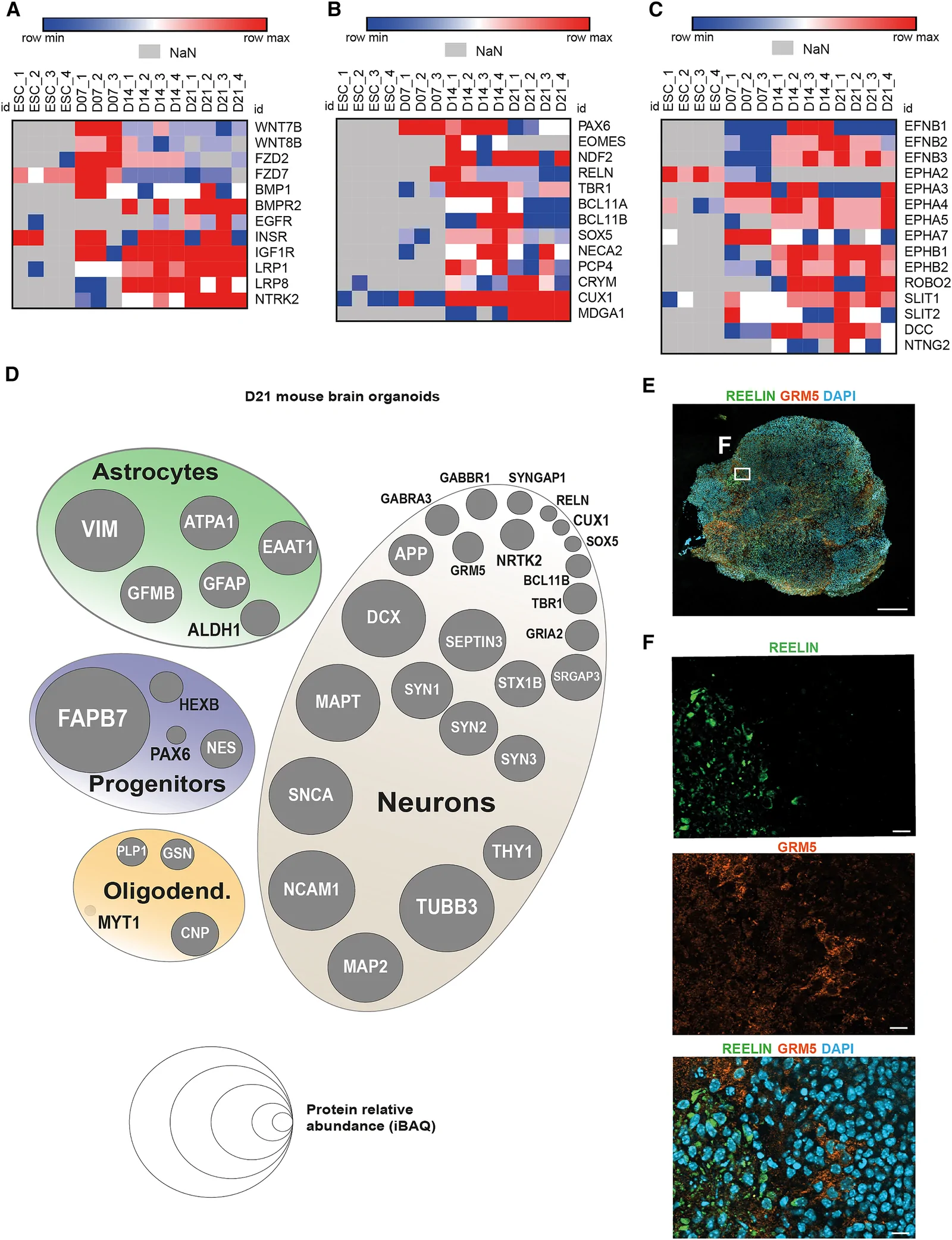
Evolution of the proteome during the development of brain organoids and the relative abundance of known brain proteins in D21 organoids (A) Heatmaps report the level (low: blue, average: white, high: red) of protein expression across samples for a selection of morphogens, growth factors and cognate receptors. The heatmap was generated using Morpheus (the Broad Institute, MIT). “Not a Number” (NaN) values are in gray. (B) Heatmap of protein expression for cortical layer markers. (C) Heatmap of protein expression for chemoattractive/chemorepulsive molecules. (D) Proteins known to be expressed in neural progenitors, neurons, astrocytes, and oligodendrocytes are represented as concentric circles proportional to their relative abundance (iBAQ) in D21 organoids. (E) D21 organoid cryosections were stained with REELIN (green), GRM5 (orange), and DAPI (blue) and imaged with a fluorescence microscope using the apotome mode. Scale bars, 200 μm. (F) High-magnification images from the inset drawn in E. Scale bars, 10 μm.

One question is, therefore, what drives the growth of organoids? During neural induction, the only exogenous growth factor protein is insulin, present in the KSR,^9^ and we detected the insulin receptor (INSR) in all developmental stages (although it was not detected in all samples). Thus, insulin transducing via the INSR might be the primary signaling pathway responsible for organoid growth. Insulin might work in conjunction with lipids since the KSR contains lipid-rich albumin,^36^ and we found that brain organoids expressed the apoE/lipoprotein receptor LRP, known to mediate lipid uptake in the brain.^37^ In the developing cortex, stem cells integrate a combination of morphogens, growth factors, and intrinsic cues to produce cells in a timely, invariant order: PAX6+ neural progenitors are generated first, followed by EOMES/TBR2+ progenitors. Next, these progenitors progressively generate neurons of deep, then upper-layer identities.^38^^,^^39^ As shown in Figure 6B, the timing of appearance of protein markers of cell identity in brain organoids reflected quite well the *in vivo* situation: PAX6 appeared first (from day 7), EOMES/TBR2 was exclusively expressed at day 14, and, for neurons, REELIN (a marker of Cajal-Retzius neurons) appeared first, followed by TBR1 (layer VI), deep layers (BCL11A/B and SOX5), and upper layers (CRYM and MDGA1). This suggests that the temporal specification of corticogenesis is quite well maintained in mouse brain organoids.

In the developing brain, neurons acquire a complex repertoire of chemoattractive/chemorepulsive molecules, including EPHRIN receptors and ligands^40^ and SLIT/ROBO.^38^ These intercellular communication systems position cells, delimit tissue boundaries, and guide navigating axons toward their targets. We found that brain organoids expressed the transmembrane EPHRINS B1-B3, as well as some EPHA and EPHB receptors, but not the GPI-anchored Type A EPHRINS (Figure 6C). As reported by Fernandez-Alonso and colleagues,^41^ we observed that EPHA2 was enriched in pluripotent cells (Figure 6C). ROBO2 and SLIT1, and their regulators NETRIN and DCC, were also detected by proteomics (Figure 6C). Consistent with these findings, brain organoids were enriched in proteins composing the KEGG pathways for “axon guidance” (FDR: 8.2E−15) and “cell adhesion molecule interactions” (FDR: 2.3E−07), in particular for the neural system (Figure S7).

Thus, collectively, brain organoids harbor a complex repertoire of proteins involved in neurodevelopment.

### Determination of the relative abundance of brain-specific proteins in brain organoids

Next, we sought to determine the relative abundance of proteins in brain organoids and took advantage of intensity-based absolute quantification (iBAQ) values.^42^ As previously described for human brain organoids,^12^ we represented the abundance of proteins known to be selectively enriched in defined brain cell types as circles whose size is proportional to their iBAQ values (Figure 6D). We focused on day 21 organoids and detected an abundant expression of the radial glia marker FABP7 (median iBAQ value: 1.11E+09) while the pan-neural progenitor marker NESTIN was less expressed (median iBAQ value: 5,54E+06). This supports the view that in D21 organoids, the most represented type of neural progenitors is radial glia, the cell type that generates neurons.^43^ The proteome of D21 brain organoids also contained the astrocyte markers VIMENTIN (median iBAQ value: 1.83E+08) and GFAP (8,51E+06) (Figure 6D). There was also an abundant expression of neuronal proteins, including cytoskeletal proteins (TUBB3; median iBAQ value: 4,00E+08, MAP2; 7,54E+07, and MAPT: 1,19E+08). We also detected proteins involved in synaptic transmission, including SYNAPSINs 1, 2, and 3, the ionotropic and metabotropic receptors for glutamate (GRM5 and GRIA2, respectively) and for GABA (GABBR1 and GABRA3, respectively) (Figure 6D). The neurotrophin receptor NRTK2/TRKB, which regulates synaptic strength,^44^ was also expressed. Consistent with these findings, brain organoids expressed several components of the KEGG pathways for “glutamatergic synapse” (FDR: 5.7E−10) and “GABAergic synapse” (FDR: 1.1E−09) (Figure S8). Not surprisingly, and as indicated by the relative size of circles, all of these receptors were less abundant than neuronal filaments. We also detected markers of layers of the cerebral cortex^18^^,^^38^: REELIN for Cajal-Retzius neurons; TBR1, SOX5, and BCL11B (also known as CTIP2) for deep layers, and CUX1 for upper-layer neurons (Figure 6D). Immunofluorescence experiments confirmed that REELIN and GRM5 were expressed in brain organoids (Figure 6E), as well as TBR1 and CTIP2 (Figures 1E and S1). However, brain organoids failed to recapitulate the laminar organization of the cerebral cortex (Figures 1D, 1E, and S1), as described by others.^4^ The spatial disorganization was not restricted to cortical layers since newly specified axons, labeled with an antibody to TRIM46,^45^ seemed randomly distributed in organoids while they adopted a parallel organization in the NBB (Figure S9). The typical markers of the oligodendrocyte lineage, PLP1 (median IBAQ value: 1.84E+06), GSN (2.31E+06), and CNP (7.47E+06) were less abundant than those of radial glia, neurons, and astrocytes, indicating an underrepresentation of this cell type (Figure 6D). By contrast, we did not detect microglia-enriched proteins (such as TMEM119, P2Y12, and SALL1^46^) or blood vessels (CD31/PECAM1), consistent with RNA-seq data.

### Correlation between the proteomes of D21 brain organoids and newborn brain

As a gold standard for brain organoids, we profiled the proteome of NBB. Our transcriptomic analyses indicated that, among the organoid time points profiled, D21 organoids are the most transcriptionally similar to the newborn brain. We therefore selected D21 organoids for direct proteomic comparison with NBB. D21 organoids were included in a second round of proteomics experiments to prevent technical artifacts that could bias their comparison with NBB samples (Figure 7A and Table S22). PCA showed a good proximity between D21 organoids from the first and second rounds of proteomics, arguing for good technical reproducibility (Figure S10). The PCA further showed that organoids progressively approached the NBB (Figure S10), in agreement with the PCA performed on RNA-seq data (Figure 2). Next, we sought to identify the differentially expressed proteins (DEPs) between D21 brain organoids and NBB. Out of 6685 quantified proteins, there were only 141 DEPs (Figure 7B). Strikingly, the 106 DEPs enriched in the NBB were all associated with BP terms linked to blood (Figure 7B), including proteins of the hemoglobin complex (Figure 7B). Hemoglobin proteins are produced by non-neural cells, erythrocytes, which travel through the brain capillaries,^47^ which explains their absence in organoids. PECAM1 was absent in organoids but present in the NBB, and this was confirmed by immunofluorescence (Figure S11). We cannot conclude for microglia since we did not detect their signature proteins TMEM119, P2Y12, and SALL1 in the NBB. By contrast, there was no BP term associated with the 35 DEPs enriched in the D21 brain organoids.

**Figure 7:**
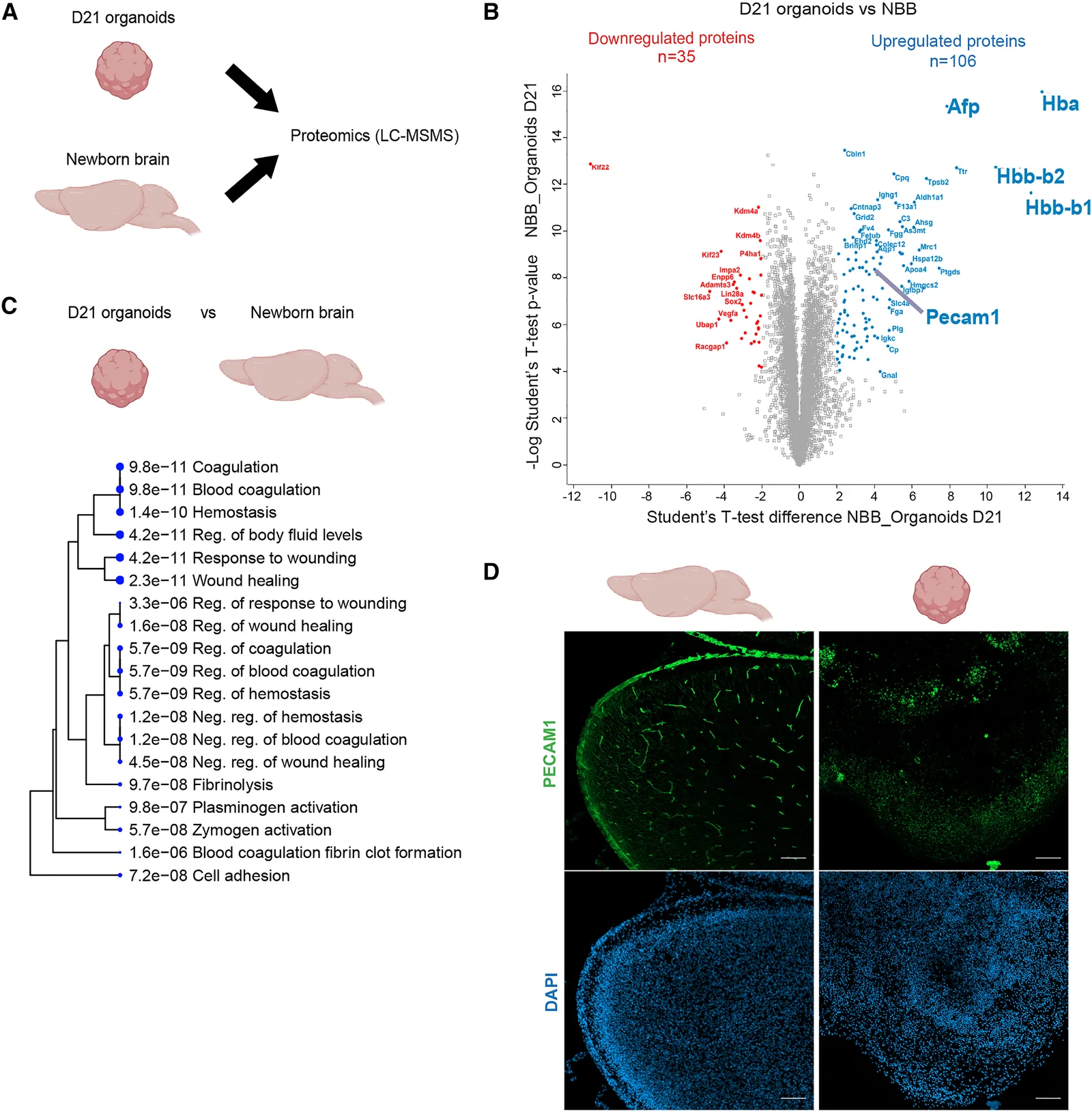
Comparison of the proteomes of brain organoids and newborn brain (A) NBB and brain organoids (at day 21) were profiled by proteomics. The figure was created with BioRender. (B) Volcano plots of differentially expressed proteins (DEPs) between NBB and D21 organoids. Proteins significantly downregulated are indicated in red, and upregulated proteins are in blue. (C) Clustergram of biological process (BP) terms found enriched in NBB compared to D21 brain organoids. (D) Immunostaining of PECAM1/CD31 (green) in P1 mouse brain (left) and D21 organoids (right). Nuclei were stained with DAPI. Scale bars, 100 μm. Extended version Figure S11.

Thus, the proteomes of D21 organoids and the NBB are very similar. In particular, D21 organoids express proteins specific to the different cell types composing the newborn brain (but not blood vessels) as well as a complex repertoire of proteins involved in synaptic transmission.

### Correlation between differentially expressed RNAs and proteins during developmental transitions and network analysis

Next, we sought to determine whether changes in RNA expression are converted into proteins during brain organoid development.

First, we noticed that out of the 15696 gene products quantified by RNA-seq, 13185/15696 (84%) were protein-coding (Figure S12A). On the other hand, proteomics on mESC-derived brain organoids detected a high number of 5,971 proteins (Figure S12B). We reasoned that since changes in RNA and proteins were both quantified by a log2-fold change, we could directly compare their variations using a Pearson’s correlation. We found a “nomenclature match” between 97% of proteins and RNAs (not shown). In all three developmental transitions (from ESCs to D7, D7 to D14, and D14 to D21), the Pearson’s coefficient *r* > 0.67 (Figure 8A), indicating a highly positive correlation between protein and RNA variation during the genesis of mouse brain organoids. The correlation was rather stable during development (*r* = 0.68 for ESC to D7 organoids, *r* = 0.69 for D7 to D14 organoids, and *r* = 0.72 for D14 to D21 organoids). Visualizing the changes in RNA and protein expression for a series of genes involved in development confirmed the largely parallel evolution of RNAs and proteins during brain organoid genesis (Figure 8B). Thus, during mouse brain organoid development, most changes in mRNA expression were translated into proteins. This contrasts with developing gastruloids, where correlations between the proteome and transcriptome fluctuate.^48^

**Figure 8:**
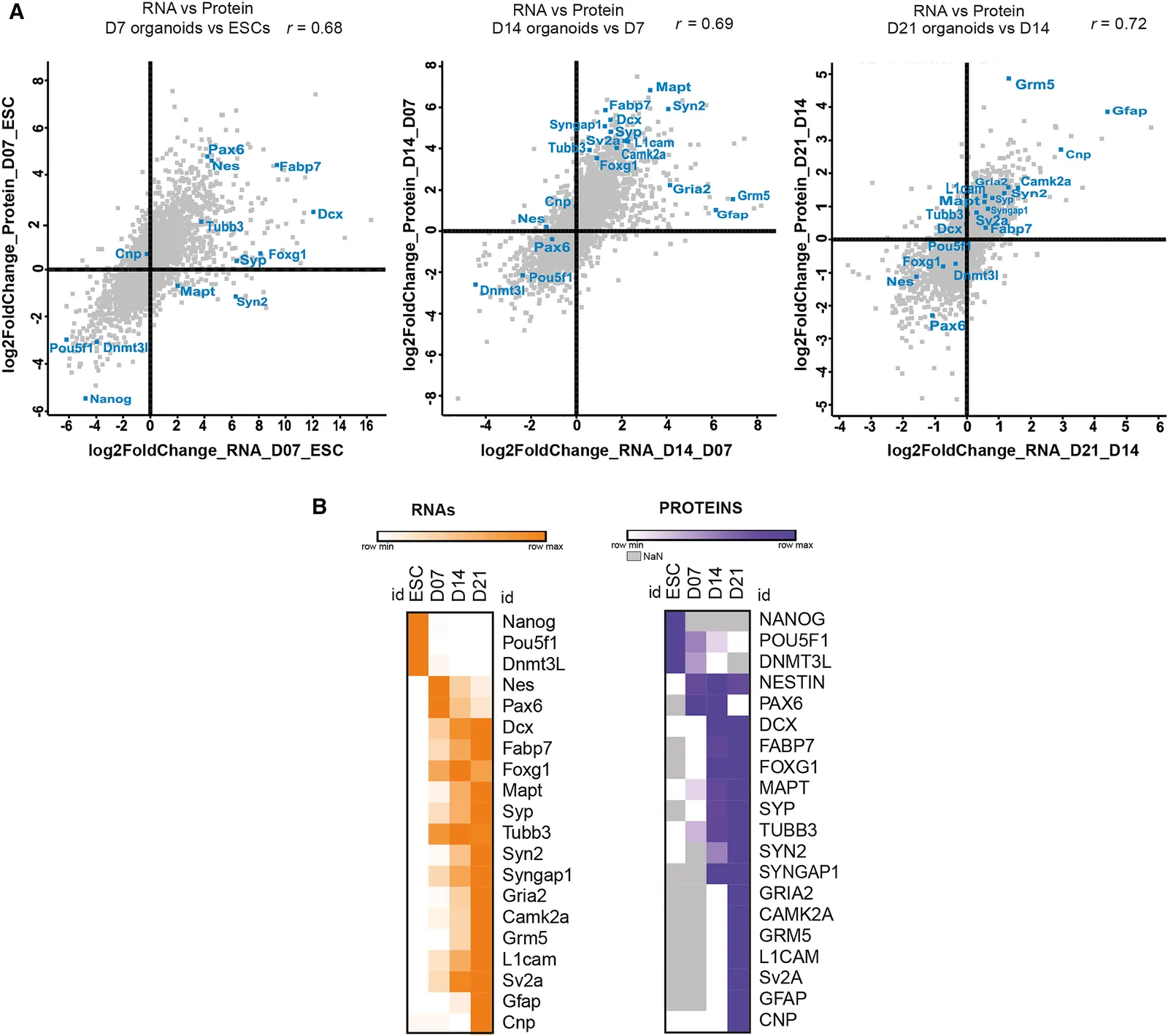
High correlation between differentially expressed RNAs and proteins during developmental transitions in brain organoids (A) Log2-Fold changes in protein (Y axis) and gene expression (X axis) were plotted during developmental transitions from mESC to D7 organoids, from D7 to D14 organoids, and from D14 to D21 organoids, and Pearson’s correlation coefficients r were calculated. (B) Heatmap reporting the levels of RNA (low: white, high: orange) and protein (low: white, high: purple, not a number (NaN) values in gray) expression across samples for cell fate markers. The heatmap was generated using Morpheus (the Broad Institute, MIT).

Finally, we sought to identify some potential orchestrators of the regulatory protein network taking place during organoid development. Analysis of DEPs between D21 organoids and ESCs using the “TF.Target.TRANSFAC.and.JASPAR.PVM” interface on ShinyGO revealed an enrichment for targets of the transcription factor Tead2 (Figure S13). In brain organoids, Tead2 mRNA peaked at days 7–14 (not shown), a timing that is consistent with Tead2’s established role in neural tube closure.^49^ The microRNA miR-124a, a brain-enriched microRNA involved in neurodevelopment,^50^ was also found as a potential regulator (Figure S14). Functional enrichment analysis of protein-protein interaction (PPI) networks using PPI-STRING (Search Tool for Recurring Instances of Neighbouring Genes)^51^ revealed a series of proteins acting as nodes (Figure S15). The three most significant proteins, DLG4 (ENSMUSP00000104229; FDR:8.0E−44), SYNAPSIN1 (ENSMUSP00000080568; FDR: 5.1E−41), and GRIN1 (ENSMUSP00000028335; FDR: 4.6E−41), are well known for their roles in synaptic transmission.

Thus, during the development of mouse brain organoids, most of the mRNA changes were translated into proteins. Tead2 and miR-124a are potential orchestrators of the associated protein regulatory network where DLG4, SYNAPSIN1, and GRIN1 acted as nodes.

## Discussion

Many brain diseases, including those that clinically manifest at adult age, such as Huntington’s and Alzheimer’s diseases, have a neurodevelopmental origin.^52^^,^^53^ During the last century, insights into normal and pathological neurodevelopment have been gained from a variety of biological models, including C. elegans, Drosophila, mouse, and human samples. In this study, we present compelling evidence that mouse brain organoids are an attractive alternative model.

*In vitro* models that differentiate embryonic stem cells into brain cells in Petri dishes (in two dimensions, 2D) reproduce some cardinal features of the *in vivo* brain, including the sequential generation of cortical layer markers.^18^^,^^54^ These models are also useful to discover genes involved in neurodevelopment^55^ and human specification.^56^ Concomitant with these 2D models, the Sasai group developed brain organoids,^1^ which were later improved by other groups to study microcephaly,^57^^,^^58^ and further complexified into brain assembloids.^59^

Brain organoids have been generated from pluripotent cells of several species, including mouse,^1^ chimpanzee and macaques,^60^ and human.^1^^,^^57^^,^^60^ The developmental timing of the brain generated from pluripotent cells is shorter for the mouse compared to primates.^61^ Brain organoids capture these species differences.^3^^,^^60^ The reproduction of human-specific features (including a complex genetic background) likely explains why most studies are being conducted on human organoids. However, mouse organoids represent an alternative model for studying neurodevelopment and function because they have a shorter generation time than human organoids. In addition, similarly to human cells, mouse ESCs can be specified into region-specific brain organoids,^1^^,^^3^^,^^9^ and they reproduce the cardinal features of *in vivo* development, including the sequential generation of cortical neurons.^1^ Here, we further show that mouse brain organoids contain NSCs that can be expanded, frozen, thawed, and differentiated into neurons and astrocytes *in vitro*.

One likely advantage of mice over humans is their defined genetic background. By crossing C57Bl/6J laboratory mice with wild-derived inbred strains to generate F1 hybrid epiblast stem cells, Medina-Cano et al. have revealed the *cis*-acting transcriptional regulatory changes in mouse brain organoids.^4^ Here, we have added a supplemental layer to our understanding of gene regulation in mouse brain organoids by showing that the transcriptome of mouse brain organoids progressively approaches that of the mouse newborn brain. Gene Ontology analysis revealed an enrichment in the same most significantly enriched neural terms as the NBB, while showing an absence of non-neural terms. This contrasts with our previous work on *in vitro* cortex derived from mESCs in a Petri dish, where we noticed an expression of endoderm and mesoderm genes, suggesting the presence of (unwanted) cell types of other developmental origins in the 2D model.^19^

Here, we have further observed that mouse brain organoids robustly reproduced two features of gene regulation important for setting a neural identity: alternative splicing and polyadenylation site usage,^14^^,^^15^ which, overall, resulted in exon skipping and 3′UTR lengthening, respectively. Thus, brain organoids might represent an attractive model to study these important gene regulatory mechanisms.^14^^,^^15^ Genes whose transcripts experienced exon alternative usage (whether it related to exon inclusion or exclusion) were related to neurodevelopment. More unexpectedly, for extending transcripts, in addition to genes known to play a role at the synapse, such as *Homer2*, we found an enrichment for the ubiquitin-proteasome pathway. The ubiquitin-proteasome pathway is known to be essential for synaptic plasticity by maintaining protein homeostasis via protein clearance,^62^ but whether the act of 3′UTR lengthening plays a role remains to be defined.

We also observed that during the generation of brain organoids, most of the changes in RNA levels are translated into changes at the protein level. We detected a high number of proteins (5971, all stages included) and observed a profound remodeling of the protein landscape during developmental transitions from pluripotency to neural induction (D7 organoids) and during the neural maturation of organoids (from day 7 to day 21). Notably, the proteome of brain organoids at day 14 indicates a certain degree of maturity, in agreement with calcium recordings performed on mouse brain organoids generated according to a similar protocol.^1^ Ciarpella and co-workers^63^ also reported the generation of brain organoids from mouse neural stem cells and evidenced a functional network maturation after less than 5 weeks.

Brain organoids also expressed a selection of proteins of the Ephrins/Ephrin-receptors and Slit-Robo families, which mediate intercellular communication. This finding might explain why, upon grafting, the axons of transplanted neurons generated *in vitro* from pluripotent stem cells navigate and connect with the appropriate targets in the host brain.^18^^,^^54^^,^^64^^,^^65^ However, the combination of Ephrins/Ephrin-receptors/Slit-Robo is cell type-specific, and we performed bulk proteomic experiments on brain organoids that contain a myriad of cell types. Single-cell proteomics could help establish whether the combination of Ephrins/Ephrin-receptors/Slit-Robo in brain organoids is cell type-specific.

Finally, thanks to iUBAQ values, we have determined the relative abundance of proteins and uncovered that most of the synaptic components are present in organoids at day 21. In contrast to the proteome of human brain organoids, which did not contain REELIN,^12^ a protein important for neuronal migration in the cerebral cortex,^66^ REELIN was found in the proteome of mouse brain organoids. We also detected the presence of the REELIN receptor LRP8/ApoER2, as well as proteins known to be enriched in deep and upper-layer cortical neurons (TBR1, BCL11B, SOX5, and CUX1). By immunofluorescence, we confirmed the presence of REELIN, TBR1, and CTIP2.

We also detected GRM5 and several other G Protein-Coupled Receptors, a class of receptors that represent one-third of approved drugs.^67^ Thus, mouse brain organoids might be a platform for drug discovery and testing.

To conclude, our study indicates that mouse brain organoids represent a relevant model to study neurodevelopment in mammals, particularly gene regulation, and brain-enriched receptors such as GABAergic and glutamatergic receptors in a neonatal-like environment.

### Limitations of the study

Our conclusion is based on bulk transcriptomics and proteomics. A far-reaching comparison of brain organoids to the *in vivo* brain requires single-cell (sc) experiments. First, sc experiments could inform about the cellular composition of brain organoids. Using sc-RNA-seq, brain organoids derived from mouse EpiSCs were found to recapitulate the cell-type diversity of the *in vivo* brain (neural progenitors, neurons, astrocytes, oligodendrocyte progenitor cells, and ependymal cells).^4^ Next, sc experiments coupled to splicing and APA analyses could inform whether mRNA isoforms are cell type-specific. Notably, it will be interesting to determine whether mouse brain organoids recapitulate the cell-type specificity of 3′UTR isoforms of the developing mouse embryo.^68^ Last, the sc proteome could inform about the correlation between protein abundance and mRNA expression at single-cell resolution. A recent study suggests that in the developing mouse brain, the correlations between the proteome and transcriptome are cell-type dependent:^69^

We confirmed that mouse brain organoids do not mimic the spatial organization of the *in vivo* brain. In particular, they failed to reproduce the laminar organization of the cerebral cortex and the parallel organization of newly specified axons. Positioning cues might be missing and/or their effect altered by the centrifugal force imposed by orbital shaking.^70^

We found that mouse brain organoids lack cell types of other developmental origins (blood vessels), as observed using sc RNA-seq.^4^

Brain organoids also lack the sensory inputs coming from other brain parts (which could be somehow mimicked using assembloids^59^), as well as those arising from other body parts and traveling through the blood-brain barrier, such as microbiota-derived signals. To tackle the influence of these extrinsic cues on brain organoid formation, the mouse model is likely more appropriate than the human one, since *in vivo* samples serving as gold standards are less rate-limiting.

We observed that the proteome of brain organoids comprises several morphogens but did not provide evidence for their secretion. However, morphogens were retrieved in the secretome of human brain organoids.^71^

We did not perform functional studies. However, it was shown that mouse brain organoids developed using the original protocol that inspired ours display spontaneous calcium activities and cell synchronicity over long distances.^1^ Using multielectrode arrays, it was further shown that mouse brain organoids (developed using a different protocol from ours) organize into neural networks, whose topologies are different between dorsal and ventral forebrain organoids.^72^

## Resource availability

### Lead contact

Requests for further information and resources should be directed to and will be fulfilled by the lead contact, Tristan Bouschet (tristan.bouschet@igf.cnrs.fr).

### Materials availability

This study did not generate new, unique reagents.

### Data and code availability

• The RNA-seq data for mouse brain organoids have been deposited in the GEO database with the identifiers GSE324877 and GSE325582.
• The mass spectrometry proteomics data have been deposited in the ProteomeXchange Consortium via the PRIDE (Proteomics Identifications) partner repository^73^ with the dataset identifier PXD075676.
• This paper does not report original code.
• Any additional information required to reanalyze the data reported in this paper is available from the lead contact upon request.

## Acknowledgments

We thank the members of the Arnaud lab, Journot lab, and the Proteomics facility for feedback and discussion. We thank Pedro Miura for helpful advice on alternative polyadenylation. We thank Anne Guillou for training and access to the cryostat facility (RHEM), and Marie-Pierre Blanchard and Jérémy Galtier from the Light Microscopy facility of Biocampus Montpellier Ressources Imagerie (MRI) for training and access to the confocal microscope. We thank Nicolas Boucharel (IGF) for performing mycoplasma assays and Céline Galindo for preparing coverslips. We thank Nathalie Bouquier and Julie Perroy (IGF) for providing the GRM5 antibody, and Charles Hong (Michigan State University) for DMH1-HCl. We thank Luc Forichon (RAM-iExplore) and Xavier Bonnefont (IGF) for their help with newborn C57BL/6J mice, and Anne Guillou and Valentin Garcia (Réseau d'Histologie Expérimentale de Montpellier) for including brains. We thank members of the iGReD Bioinformatics Platform (BIM) for their technical assistance and discussions on RNA sequencing analysis. We thank Oana Vigy (FPP) for depositing the mass spectrometry proteomics data to the ProteomeXchange Consortium via the PRIDE partner repository. The Orbitrap Exploris 480 mass spectrometer used at the Montpellier Proteomics Platform (PPM, BioCampus) for proteomics analysis was co-financed by the 10.13039/501100008530European Regional Development Fund (10.13039/501100008530ERDF) and the Occitanie region. PPM is a member of the national Proteomics French Infrastructure (ProFI UAR 2048) supported by the 10.13039/501100001665French National Research Agency (ANR-24-INBS-0015, Investments for the future F2030). This work was supported by the Grant CORGI (ANR-23-CE12-0017, to P.A. and T.B.).

## Author contributions

Conceptualization, B.M., P.A., S.U., F.C., and T.B.; methodology, B.M., P.A., S.U., F.C., and T.B.; investigation, A-C. F., C. G. G., K.E.K., S. P., S.U., F.C., and T.B.; writing of the original draft, A-C. F., and T.B.; writing: review and editing, A-C. F., B.M., P.A., S.U., F.C., and T.B.; funding acquisition, P.A. and T.B.; resources, P.A., S.U., F.C., and T.B.; supervision, S.U., F.C., and T.B. All authors read and approved the final manuscript.

## Declaration of interests

The authors declare no competing interests.

## STAR★Methods

### Key resources table

**Table:** 

REAGENT or RESOURCE	SOURCE	IDENTIFIER
**Antibodies**		

Mouse monoclonal anti-NESTIN (Rat-401)	Santa-Cruz	Cat# sc-33677; RRID: AB_627995
Rabbit polyclonal Anti-PAX6	Covance	Cat# PRB-278P; RRID: AB_291612
Mouse monoclonal anti-NANOG	BD Pharmingen	Cat# 560259; RRID: AB_1645261
Rabbit monoclonal anti-POU5F1	Cell Signaling	Cat# 2840; RRID: AB_2167691
Mouse monoclonal anti-ELAVL3	Santa Cruz	Cat# sc-515624
Mouse monoclonal anti-TUBB3	Covance	Cat# MMS-435P; RRID: AB_2313773
Rabbit monoclonal anti-TBR1	Cell Signaling	Cat# 49661; RRID: AB_2799364
Mouse monoclonal anti-REELIN	Covance	Cat# MAB5364; RRID: AB_2179313
Rabbit polyclonal anti-GRM5	Millipore	Cat# AB5675; RRID: AB_2295173
Rabbit polyclonal anti-GFAP	Dako/Agilent	Cat# Z0334; RRID: AB_10013382
Rabbit monoclonal anti-TRIM46	Synaptic systems	Cat# 377 008; RRID: AB_2905572
Chicken polyclonal anti-NeuN	Synaptic systems	Cat# 266 006; RRID: AB_2571734
Goat polyclonal anti CD31/PECAM	R&D systems	Cat# AF 3628; RRID: AB_2161028

**Biological samples**		

Mouse: newborn brain, P1, B6 (for immunofluorescence)	Réseau des Animaleries de Montpellier (RAM)-iExplore	N/A
Mouse: newborn brain, P1-P3, B6 x JF1 (for proteomics and transcriptomics)	Philippe Arnaud’s lab	N/A

**Chemicals, peptides, and recombinant proteins**		

N2 supplement	Life Technologies	17502048
B27 minus vitamin A supplement	Life Technologies	12587010
DMH1-HCL	Charles Hong	In-house synthesis
EGF	Peprotech	315–09
FGF2	Peprotech	100-18B
Y-27632	Tocris	Cat. No. 1254
ESGRO Complete PLUS Clonal Grade Medium	Merck	SF001-500P
IWP2	Tocris	Cat. No. 3533
KnockOut™ Serum Replacement -KSR-	Life Technologies	10828010

**Deposited data**		

RNA-seq data for mouse brain organoids (Days 7, 14, and 21) differentiated from B6 x JF1 mESC	This study, and Perillous et al.^17^	GEO: GSE324877, GSE325582
Proteomics data for B6 x JF1 mESC, brain organoids (Days 7, 14, and 21), and newborn brain samples	This study	PRIDE: PXD075676

**Experimental models: Cell lines**		

Mouse embryonic stem cells B6 x JF1	Philippe Arnaud’s lab Rengifo Rojas et al.^74^	N/A

**Software and algorithms**		

GENCODE vM37 basic annotation (version 2.2.1)	GENCODE	https://www.gencodegenes.org/mouse/release_M37.html
Samtools (version 1.21).	Li et al.^75^	N/A
BamCoverage (version 3.5.6)	Ramirez et al.^76^	Deeptools
Ttseq-count (version 2.0.9)	Anders et al.^77^	N/A
DESeq2	Love et al.^78^	N/A
Pheatmap R package version 1.0.13	Dave Tang	https://davetang.org/muse/2018/05/15/making-a-heatmap-in-r-with-the-pheatmap-package/
DIA-NN 1.8.2b27	Demichev et al.^79^	N/A
Perseus v1.6.15.0	Tyanova et al.^80^	N/A
DAVID	Sherman et al.^20^	N/A
rMATS turbo (version 4.3.0)	Wang et al.^21^	N/A
Apalyzer R packages (version 1.12.0)	Wang et Tian^26^	N/A
Deeptools suite (version 3.5.6)	Ramirez et al.^76^	N/A
Xcalibur 4.2 software	N/A	Thermo Fisher Scientific
ShinyGO	Ge et al.^29^	N/A
UCSC genome browser	Kent et al.^23^	https://genome.ucsc.edu/

### Experimental models and study participant details

We bred and crossed *Mus musculus* C57BL/6J and *Mus musculus molossinus* JF1 mice and collected brains from one-to three-day-old (P1-P3) newborn pups in accordance with the rules in force at the accredited animal facility (Université Clermont Auvergne, approval number D 63 1131) and European Directive 2010/63/EU. Ethical approval was not required under applicable legislation, as the experiments involved the use of postmortem samples. Mouse brains were also extracted from one-day-old C57/B6 mice obtained from the Réseau des Animaleries de Montpellier(RAM)-iExplore (approval number: B 34–172-41). The biological sex of animals was not determined. Mouse hybrid embryonic stem cells (mESCs) obtained from crosses between C57BL/6J and JF1 were previously described.^74^

### Method details

#### Differentiation of mouse embryonic stem cells into brain organoids

Mouse hybrid embryonic stem cells (mESCs) were expanded on Matrigel-coated dishes in ESGRO Complete Plus medium (Millipore) supplemented with a GSK3β inhibitor and penicillin/streptomycin as described.^19^ For cortical organoid generation, ESCs were dissociated using Accumax and resuspended at a density of 30,000 cells/mL in Cortex Medium I: DMEM/F-12/GlutaMAX supplemented with 10% KSR, 0.1 mM of non-essential amino acids, 1 mM of sodium pyruvate, 0.1 mM of 2-mercaptoethanol, 100 U/mL penicillin/streptomycin, 1 μM DMH1-HCl and 240 nM IWP-2. Cells were seeded into 96-well ultra-low-adhesion plates (Sumitomo) at 3,000 ESCs per well in 100 μL. On day 4, an additional 100 μL of Cortex Medium I was added to each well. On day 7, 4 to 6 aggregates were transferred to 6-well low-adhesion plates (Costar) and cultured in Cortex Medium 2: DMEM/F-12/GlutaMAX supplemented with N2 and B27 without vitamin A, 500 μg/mL of BSA, 0.1 mM of non-essential amino acids, 1 mM of sodium pyruvate, 0.1 mM of 2-mercaptoethanol, and 50 U/mL penicillin/streptomycin on an agitating platform (Infors Celltron) at 94 RPM. On day 14, 2 mL of medium was replaced with 2.5 mL of fresh Cortex Medium 2. Cultures were routinely tested for the absence of mycoplasma.

#### Isolation, expansion and differentiation of neural stem cells from brain organoids

Brain organoids at day 7 were rinsed with 5 mL PBS and incubated with 1 mL of Accumax (warmed at 37°C) for 5 min. Organoids were dissociated by pipetting up and down ten times with a 1-mL tip. After letting the sedimenting cell clumps settle for 1 min, the cell suspension was transferred into a new tube containing 10 mL of DMEM-F12 with 10% KSR and centrifuged for 5 min at 400g. The cell pellet was resuspended in NSC media: equal volumes of Cortex medium 2 and Neurobasal-Plus, supplemented with Glutamine (1 mM), EGF (10 ng/mL), FGF2 (10 ng/mL), and Y27632 (5 μM). 20,000 cells/mL of NSC media were plated onto plates coated with Poly-D-Lysine and Laminin.^81^ To expand NSCs, half of the media was changed with fresh NSC media (without Y-27632) every 3–4 days. When almost confluent, cells were splitted with Accumax and further expanded or frozen. To differentiate NSCs into neurons and glia, half of the media without growth factors was changed every 3–4 days.

#### Newborn brain samples

Newborn brains were collected from 1- to 3-day-old mice obtained from reciprocal crosses between C57BL/6J and JF1. Immediately after dissection, tissues were snap-frozen in liquid nitrogen and stored at −80 °C until analysis by RNA-seq and proteomics.

#### Fixation, cryoprotection, cryosectioning and immunofluorescence on brain organoids and newborn brains

Organoids were processed as described by Madeline Lancaster’s lab (https://www2.mrc-lmb.cam.ac.uk/groups/lancaster/), with slight modifications. Briefly, organoids were fixed in 4% PFA for 15 min at RT and washed three times in PBS1X for 10 min. Samples were cryoprotected by incubation in 30% sucrose overnight at 4°C. Organoids were then incubated for 15 min at 37°C and embedded in 7.5% gelatin and 30% sucrose. Blocks were frozen on dry ice pellets and stored at −80°C until sectioning. Mouse brains were extracted from one-day-old C57/B6 mice obtained from the Réseau des Animaleries de Montpellier(RAM)-iExplore (approval number: B 34–172-41). Brains were fixed overnight in 4% PFA. Fixed brains were washed twice with PBS 1× and incubated in increasing concentrations of sucrose (10, 20, and 30%) until they sank. Brains were embedded in OCT solution and 30% sucrose, snap-frozen, and stored at −80°C until sectioning.

For immunofluorescence experiments, cryosections (10 μm) were air-dried for 5 min, rinsed in PBS, and blocked/permeabilised in PBS containing 0.25% Triton X-100 and 4% horse serum for 1 h at RT. Primary antibodies were incubated overnight at 4°C. Cryosections were washed three times with PBS and incubated with secondary antibodies for 2 h at RT, both diluted in PBS containing 0.1% Triton X-100 and 4% FBS. Nuclei were stained with DAPI (1:200, 5 min at RT). The slides were mounted with a drop of mowiol and observed under a fluorescence microscope with an Apotome mode (AxioImager Z1, Zeiss) or a confocal microscope (LSM980 AIRYSCAN, Zeiss).

#### Transcriptomics

RNA Samples (brain organoids at day 7: *n* = 4, day 14: *n* = 4, day 21: *n* = 4 and newborn brain:*n* = 2) were extracted using the MACHEREY-NAGEL NucleoSpin RNA Plus kit or Zymo Quick RNA Mini Prep. After DNase treatment with the Qiagen RNase-Free DNase Set kit, the RNA samples were cleaned up with the Qiagen RNeasy Mini kit. Library preparation (NEBNext Ultra II mRNA-Seq Kit) and sequencing (on a NovaSeqX+ apparatus) were performed by IntegraGen SA according to the manufacturer’s protocol. Stranded paired-end RNA-seq reads were mapped to the mouse genome mm39, which was masked for B6/JF1 SNPs, using Hisat2 and the list of known splice sites produced with the GENCODE vM37 basic annotation (version 2.2.1; option: -no-softclip -known-splicesite-infile). The RNA-seq alignments were filtered with samtools^75^ for mapping quality and reads mapped in proper pairs (version 1.21; view option: -f 2 -q 20). The strand-specific coverages of the RNA-seq data were generated using bamCoverage (version 3.5.6; option: -normalizeUsing RPKM -filterRNAstrand forward/reverse) and visualised on the UCSC genome browser.^23^ Strand-specific coverage of replicates was overlaid using the track collection builder tool of UCSC for genome exploration.

The RNA-seq alignments were processed using htseq-count(version 2.0.9) with the GENCODE vM37 basic annotation to compute the read counts per gene (option: -s reverse). Differential gene expression analyses between samples were conducted on read counts using the DESeq2 R package, version 1.38.3.^78^ Genes were considered differentially expressed between groups when |log2-fold change| >2 with an adjusted *p*-value <0.05.

The read count data were normalised with the rlog function from the DESeq2 package before data visualisation with a heatmap (pheatmap R package version 1.0.13) and PCA (plotPCA function from the DESeq2 package).

RNA-seq data for ESCs were obtained from the Gene Expression Omnibus (GEO) database GSE244146^74^ and GSE325582 (this study). The RNA-seq data for brain organoids were deposited in the GEO database with the identifiers GSE324877 and GSE325582. In details, ESCs were obtained from GSM7807833, GSM7807834, GSM7807835, GSM7807836, GSM9608946; D7 organoids from GSM9588178, GSM9588179, GSM9608948, GSM9608949; D14 organoids from GSM9588180, GSM9588181, GSM9608950, GSM9608951; D21 organoids from GSM9588182, GSM9588183, GSM9608952, GSM9608953; NBB were obtained from GSM9588184, GSM9588185.

#### Proteomics

Samples were rinsed with PBS and kept at −80°C until analysis. Samples (ESCs: *n* = 4, brain organoids at day7: *n* = 3, brain organoids at day14: *n* = 4, brain organoids at day21: *n* = 4, and Newborn brains: *n* = 3) were digested with trypsin using the Strap (Suspension trapping) protocol. The resulting peptides were resuspended in buffer A (0.05% TFA, 2% acetonitrile) and injected for online analysis using nanoflow HPLC (RSLC U3000, Thermo Fisher Scientific) coupled to a mass spectrometer (Exploris 480). Peptides were separated on a capillary column (0.075 mm × 250 mm, Acclaim Pepmap 100, reverse phase C18, NanoViper, Thermo Fisher Scientific) following a gradient of 2–40% buffer B in 120 min (A = 0.1% formic acid, B = 0.1% formic acid, 80% acetonitrile) at a flow rate of 300 nL/min. Spectra were recorded using Xcalibur 4.2 software (Thermo Fisher Scientific) with the 120minDIA2.meth method. These instruments are checked regularly (cleaning, fluids, calibration). Spectral data were analyzed with DIA-NN^79^ 1.8.2b27 and Perseus v1.6.15.0,^80^ using the DIAGui1.4.2 R script. We used as database(s): RefProt_Mouse_2025_03_FPPCON_v1.8.2.predicted.speclib with the modifications: Carbamidomethylation, N-Term M excision. The entire experimental process was verified by K.E.K and S.U. The Exploris 480 was last visited by the manufacturer in January 2025.

### Quantification and statistical analysis

The functional annotation tool DAVID^20^ was used to identify enriched biological terms from gene lists.

Statistical analyses, plots, heatmaps, and Venn Diagrams (venneuler version 1.1–4) were generated with R version 4.5.2.

To identify differential alternative splicing events between two sample groups, the RNA-seq alignments were analyzed using the rMATS turbo^21^ (version 4.3.0) and the GENCODE vM37 basic annotation (options: -libType fr-firststrand -t paired -variable-read-length -readLength 100). Differential alternative splicing events were considered when | IncLevelDifference| >0.2 and FDR <0.05. Differential splicing event positions were loaded on UCSC in bed format in order to visualise the effect on RNA-seq coverages.

To identify alternative polyadenylation, RNA-seq alignments were treated with the Apalyzer R package (version 1.12.0).^26^ 3 ‘UTR polyadenylation sites reference was built using the GENCODE vM37 basic annotation, and significantly alternative polyadenylation events in 3’UTRs were called with the APAdiff function (option CUTreads = 10) when |RED|> 0.5 and *p*-value <0.05. To evaluate locally or globally alternative polyadenylation on RNA-seq coverage, coordinates of alternative 3′UTRs were loaded on UCSC or were treated with the Deeptools suite (version 3.5.6). Briefly, computeMatrix tools were used in scale-regions mode (-a 500 -b 500 -m 1000 --skipZeros) to calculate the strand-specific coverage of the RNA-seq per alternative 3′UTR. computeMatrixOperations was used to filter and merge the matrix produced with plus or minus strand coverage, according to the strand of the alternative 3′UTR. The corresponding heatmap was produced with the plotHeatmap tool.

Proteomic data were analyzed using the Perseus computational tool (version 1.6.15.0). Gene Ontology analysis was performed using ShinyGO^29^ with an FDR cut-off set at 0.05.

The heatmaps were generated using Morpheus: https://software.broadinstitute.org/morpheus.

## Supplemental information

Document S1. Figures S1–S15

Table S1. DESeq2_DEGs_D7_organoids_vs._ESCs

Table S2. DESeq2_DEGs_D14_vs._D7_organoids

Table S3. DESeq2_DEGs_D21_vs._D14_organoids

Table S4. DESeq2_DEGs_D21_vs._D7_organoids

Table S5. Functional_Annotation_DEGs_D7_organoids_vs._ESCs

Table S6. Functional_Annotation_DEGs_D21_vs._D7_organoids

Table S7. DESeq2_DEGs_D21_organoids_vs._ESCs

Table S8. DESeq2_DEGs_Newborn_brain_vs._ESCs

Table S9. Functional_Annotation_DEGs_D21_organoids_vs._ESCs

Table S10. Functional_Annotation_DEGs_NBB_vs._ESCs

Table S11. DESeq2_DEGs_Newborn_brain_vs._D21_organoids

Table S12. Functional_Annotation_DEGs_NBB_vs._D21_organoids

Table S13. Rmats_D21_vs._ESCs

Table S14. Rmats_NBB_vs._ESCs

Table S15. Functional_Annotation_Rmats_Skipped_exon_D21_organoids_vs._ESCs

Table S16. APAlyzer_D7_vs._ESCs

Table S17. APAlyzer_D14_vs._ESCs

Table S18. APAlyzer_D21_vs._ESCs

Table S19. APAlyzer_NBB_vs._ESCs

Table S20. Functional_Annotation_APAlyzer_3_UTR_lengthening_ES_vs._D21

Table S21. Proteomics on mESCs, mouse brain organoids at Days 7, 14, and 21

Table S22. Proteomics on NBB and mouse brain organoids at Day 21
