# Supplementary material for: The RNA and protein landscapes of mouse brain organoids
Source: iScience. 2026 Nov 5;29(11):118144. doi: 10.1016/j.isci.2026.118144 (PMC13769100; doi:10.1016/j.isci.2026.118144)
Supplement: Supplementary file 1 [file mmc1.pdf]

iScience, Volume *29*

Supplemental information

The RNA and protein landscapes of mouse brain organoids

Anne-Charlotte Fromaget, Céline Gonthier-Guéret, Khadija El Koulali, Sophie Périllous, Nuttamonpat Gumpangseth, Bertille Montibus, Philippe Arnaud, Serge Urbach, Franck Court, and Tristan Bouschet

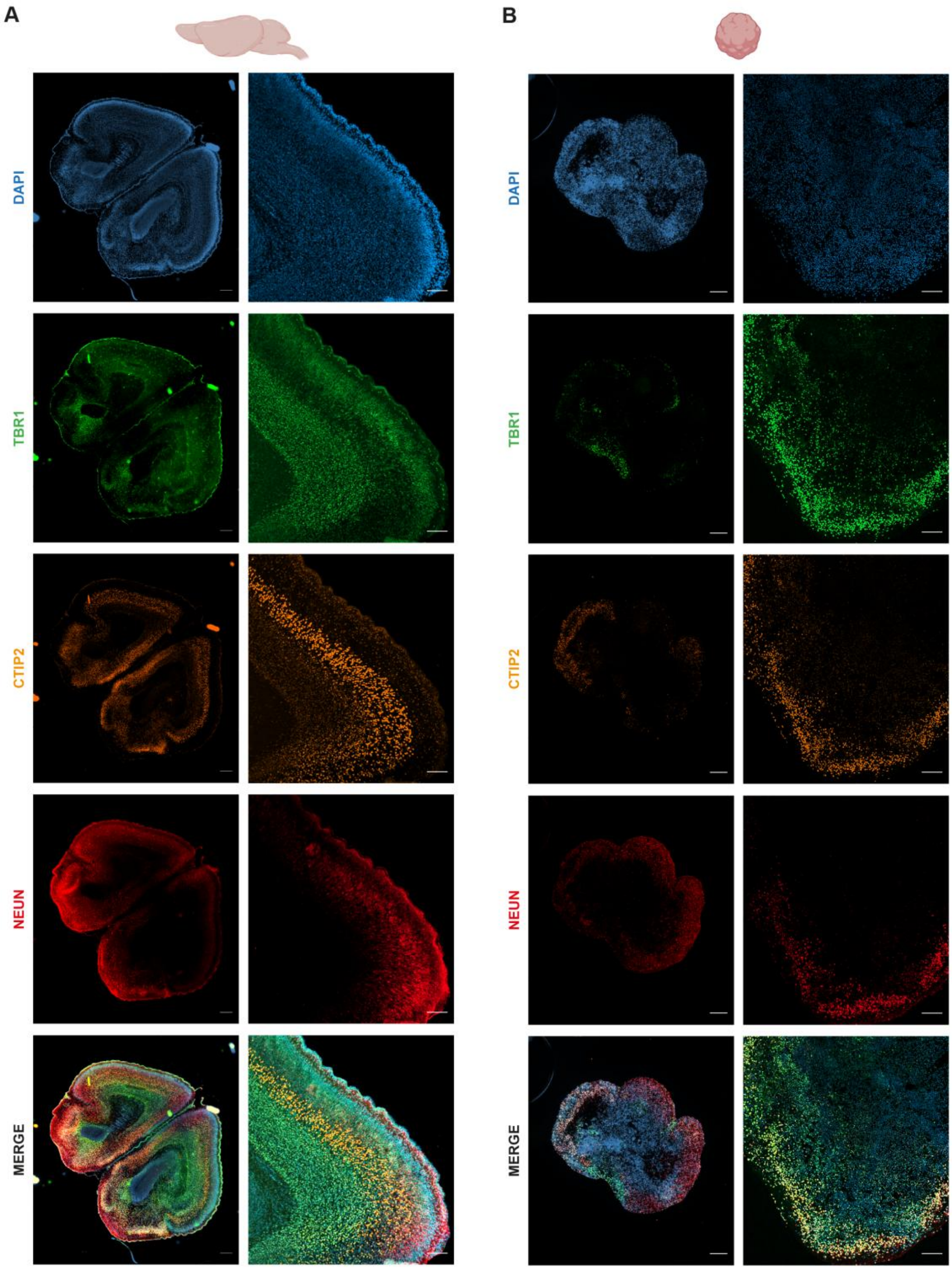

Figure S1. Absence of laminar organisation in brain organoids

(Extended version of Figure 1D and 1E)

(A) Left: expression of the cortical layer markers CTIP2 (orange) and TBR1 (green), costained with the neuronal marker NEUN (red) and DAPI (blue) in the NBB (at P1). Scale bar: 500µm. Right: higher-magnification picture centered on the cerebral cortex. Scale bar: 100µm. Part of the figure was created with BioRender.

(B) Same immunostaining as in (A) but on a brain organoid section (at day 21). Scale bars: 200µm (left), 100µm (right).

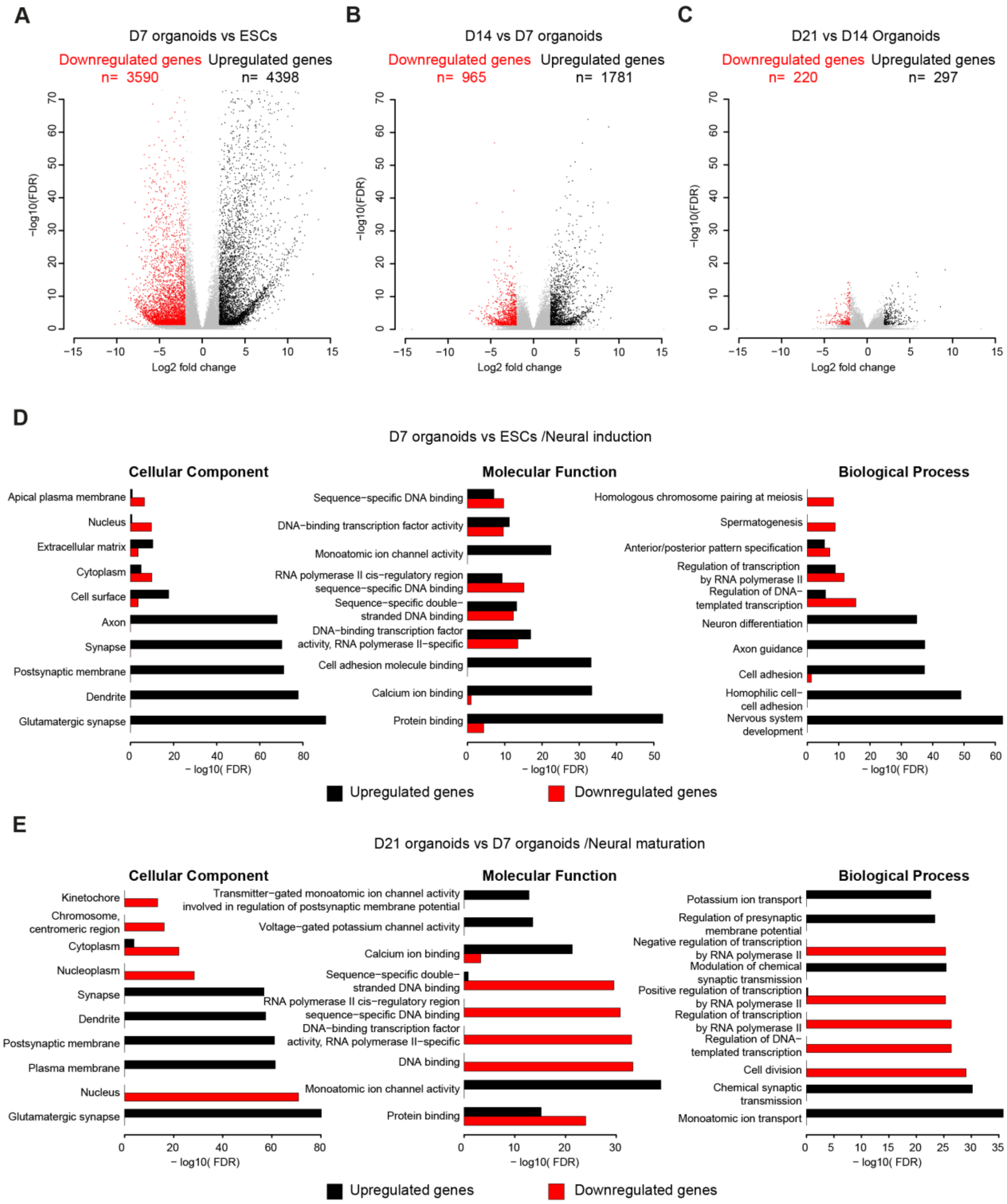

Figure S2. Functional enrichment analyses of differentially expressed genes during neural induction and maturation

(A-C) Volcano plot of the differentially expressed genes between D7 organoids and mESCs (A), D14 organoids and D7 organoids (B), D21 organoids and D14 organoids (C), illustrating the neural induction and the neural maturation of the brain organoid generation. The red and black dots, respectively, represent genes that were significantly down- or upregulated during the two developmental stages (|log2FoldChange| >2, FDR <0.05).

(D-E) Gene Ontology (GO) terms enriched for differentially expressed genes during neural induction (D7 organoids vs ESC) (D) and neural maturation (D21 vs D7 organoids) (E). For each GO term category, the five highest terms are shown for the differentially expressed gene lists. Red and black bars represent the –log10(FDR) of the downregulated and upregulated gene lists.

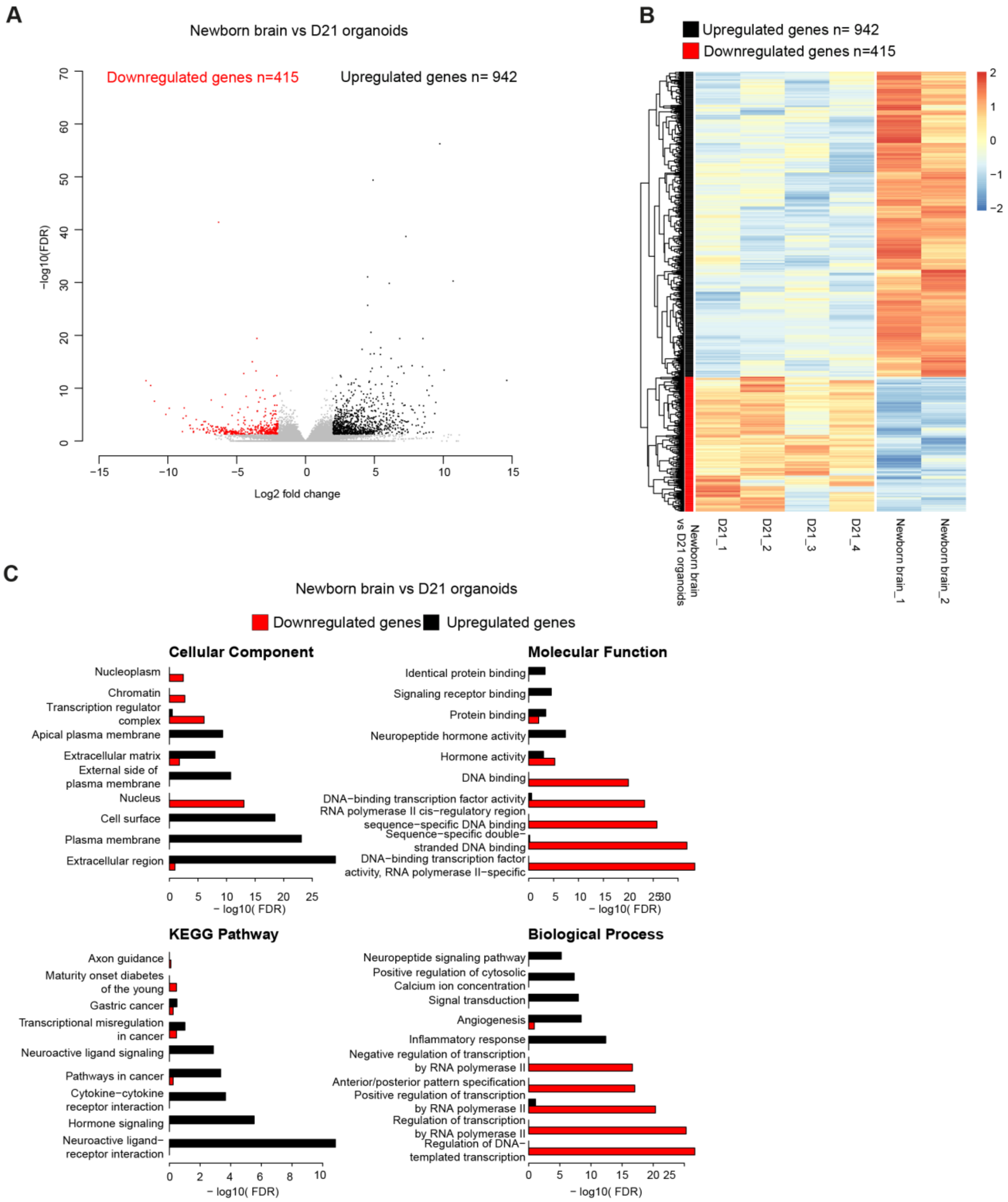

Figure S3. Analysis of differentially expressed genes between D21 organoids and newborn brain

(A) Volcano plot of the differentially expressed genes between D21 organoids and newborn brain. Brain organoid generation was associated with the underexpression of 415 genes (red) and the overexpression of 942 genes (black).

(B) Heatmap showing the centred-scaled expression levels of the differentially expressed genes between newborn brain and D21 organoids. Results of the differential analyses are shown on the left of the heatmap, with black for upregulated genes and red for downregulated genes.

(C) Functional enrichment analyses of differentially expressed genes between newborn brain and D21 organoids. For each term category, the five highest terms are shown. Red and black bars represent the –log10(FDR) of the downregulated and upregulated gene lists.

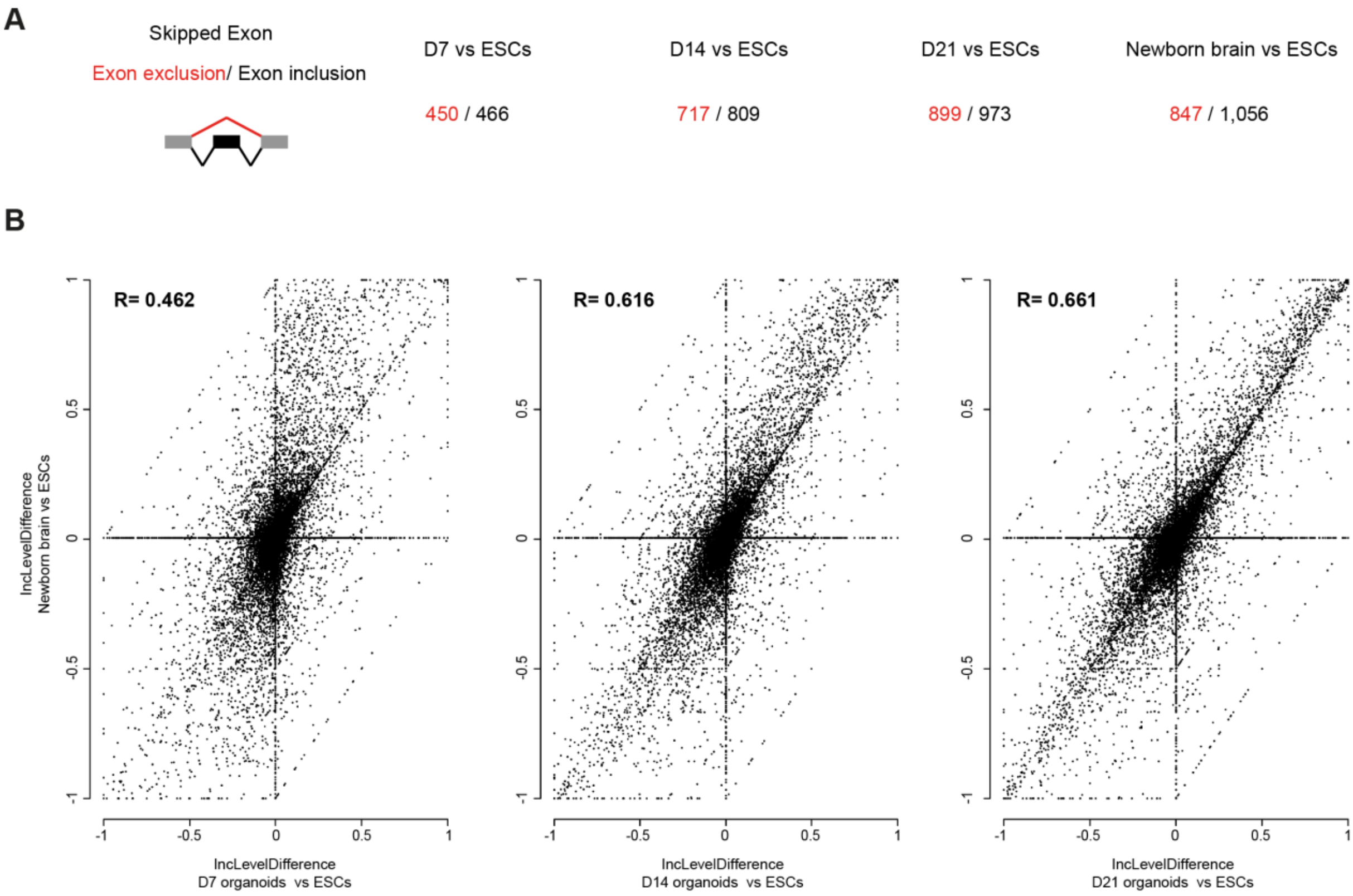

Figure S4. Evolution of alternative exon usage during brain organoid formation

(A) Evolution of alternative exon usage during organoid formation. Number of alternative exon usage events detected using R-MATS in organoid and NBB compared to mESCs (red: exclusion, black: inclusion).

(B) Pearson correlation of incLevelDifference obtained using R-MATS with the NBB and the organoids at days 7, 14 and 21.

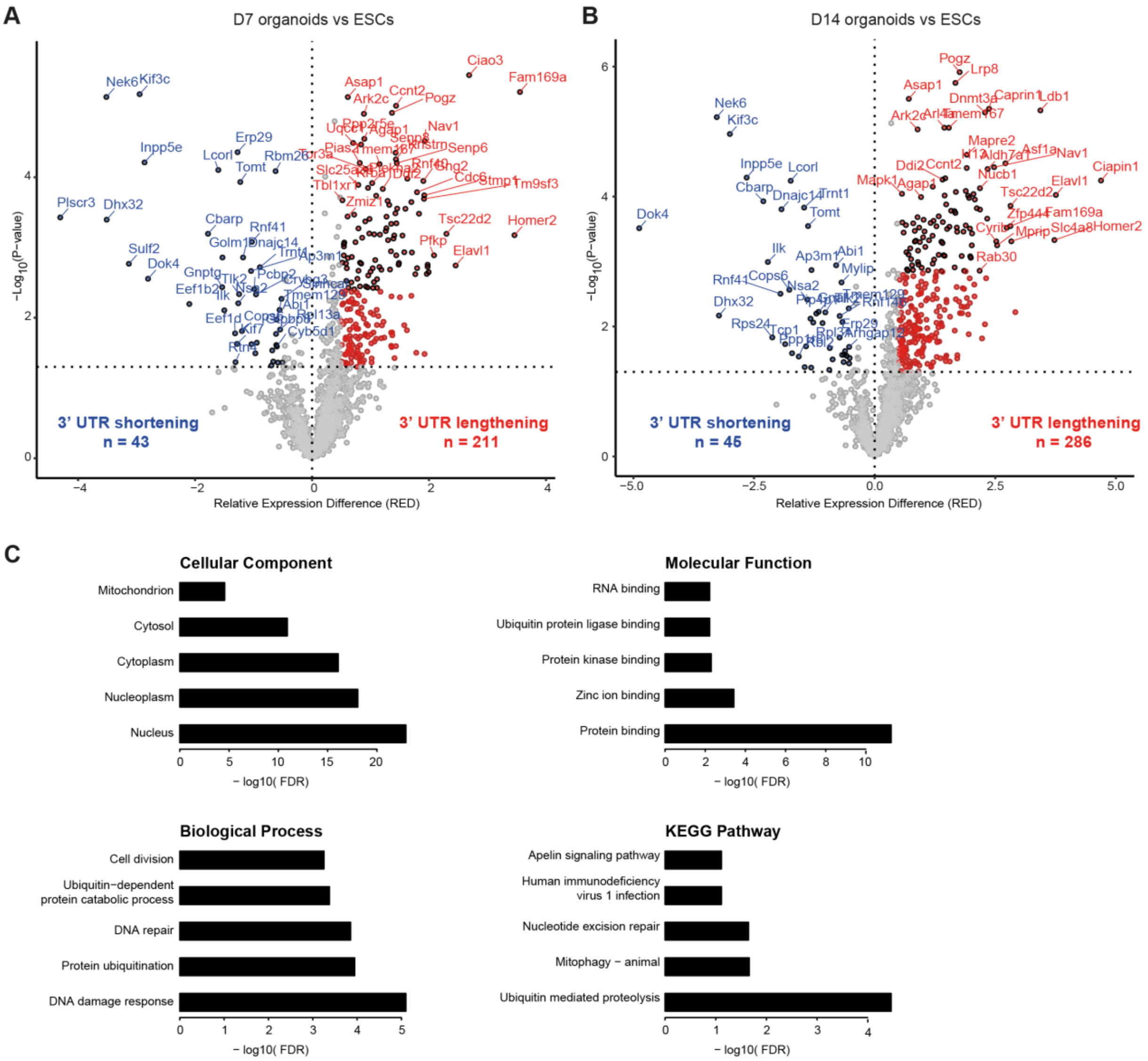

Figure S5. Evolution of alternative polyadenylation site usages during organoid formation

(A) Volcano plot of the distribution of genes identified as displaying proximal (blue) or distal (red) polyadenylation site usage in D7 organoids compared to mESCs. Genes were identified using APA analyser with the following cut-offs: -Log10(value>1.3) and RED (Relative Expression Difference) between proximal and distal polyadenylation site usage>0.5.

(B) Same as in A, but for the D14 organoids compared to mESCs.

(C) Gene Ontology (GO) was performed using DAVID on the 337 genes with 3'UTR lengthening identified by APAnalyzer during the transition from mESCs to D21 brain organoids. For each GO term category, the five highest terms are shown for the differentially expressed gene lists. Black bars represent the –log10(FDR).

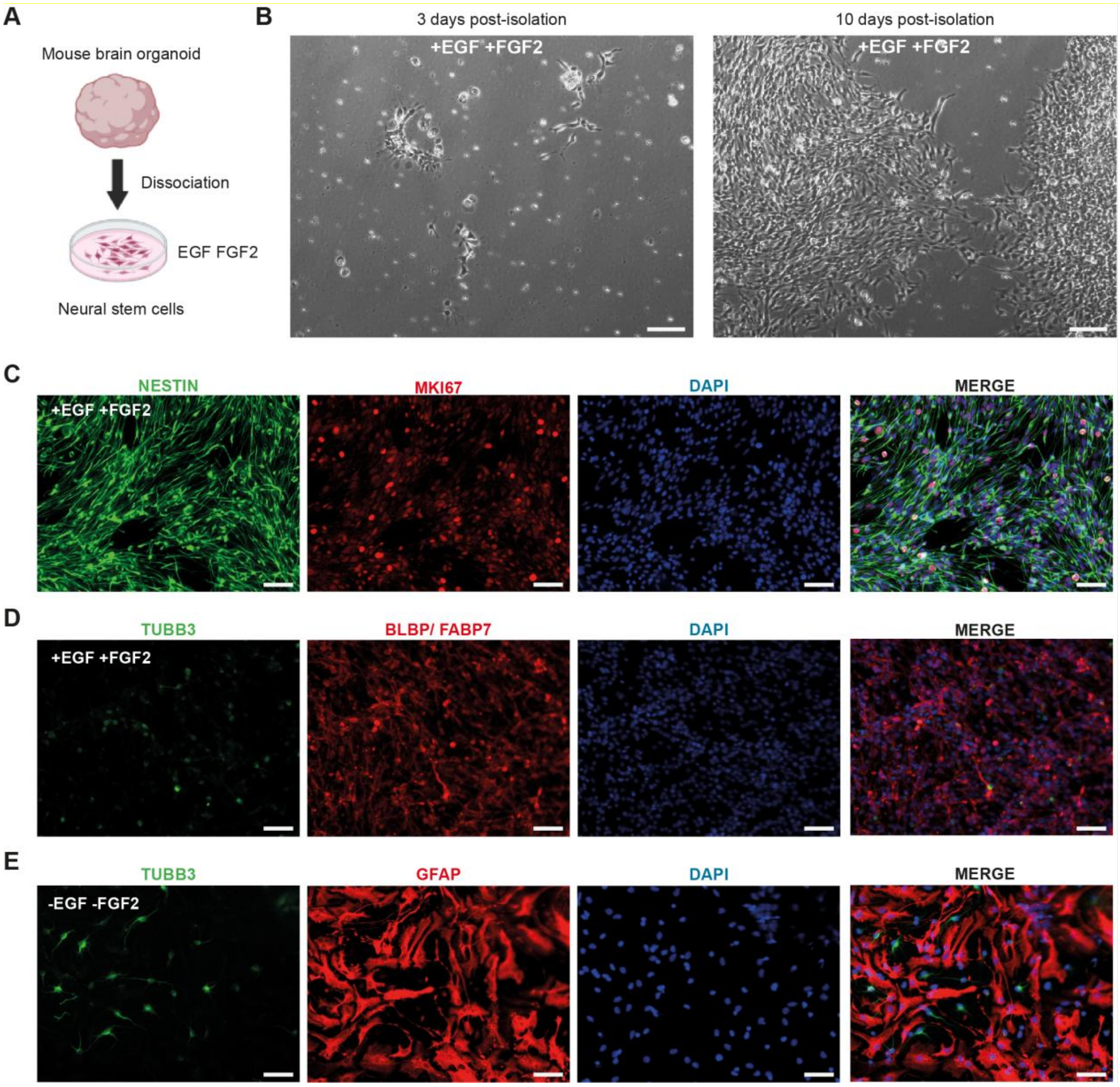

Figure S6. Generation of neural stem cells from mouse brain organoids

(A) Overview of the procedure. The figure was created using Biorender.

(B) Transmitted light pictures of cells isolated from brain organoids and cultured in the presence of EGF and FGF2 for 3 days (left) and 10 days (right). Scale bar: ~100 µm.

(C) Expression of the pan-neural progenitor NESTIN (green) and the proliferation marker MKI67 (red) in NSCs cultivated in the presence of EGF and FGF2. Scale bar: 50 µm.

(D) Expression of the neuronal marker TUBB3 (green) and the radial glia marker FABP7/BLBP (red) in NSCs cultivated in the presence of EGF and FGF2. Scale bar: 50 µm.

(E) Expression of the neuronal marker TUBB3 (green) and the astrocyte marker GFAP (red) in NSCs cultivated in the absence of EGF and FGF2. Scale bar: 50 µm.

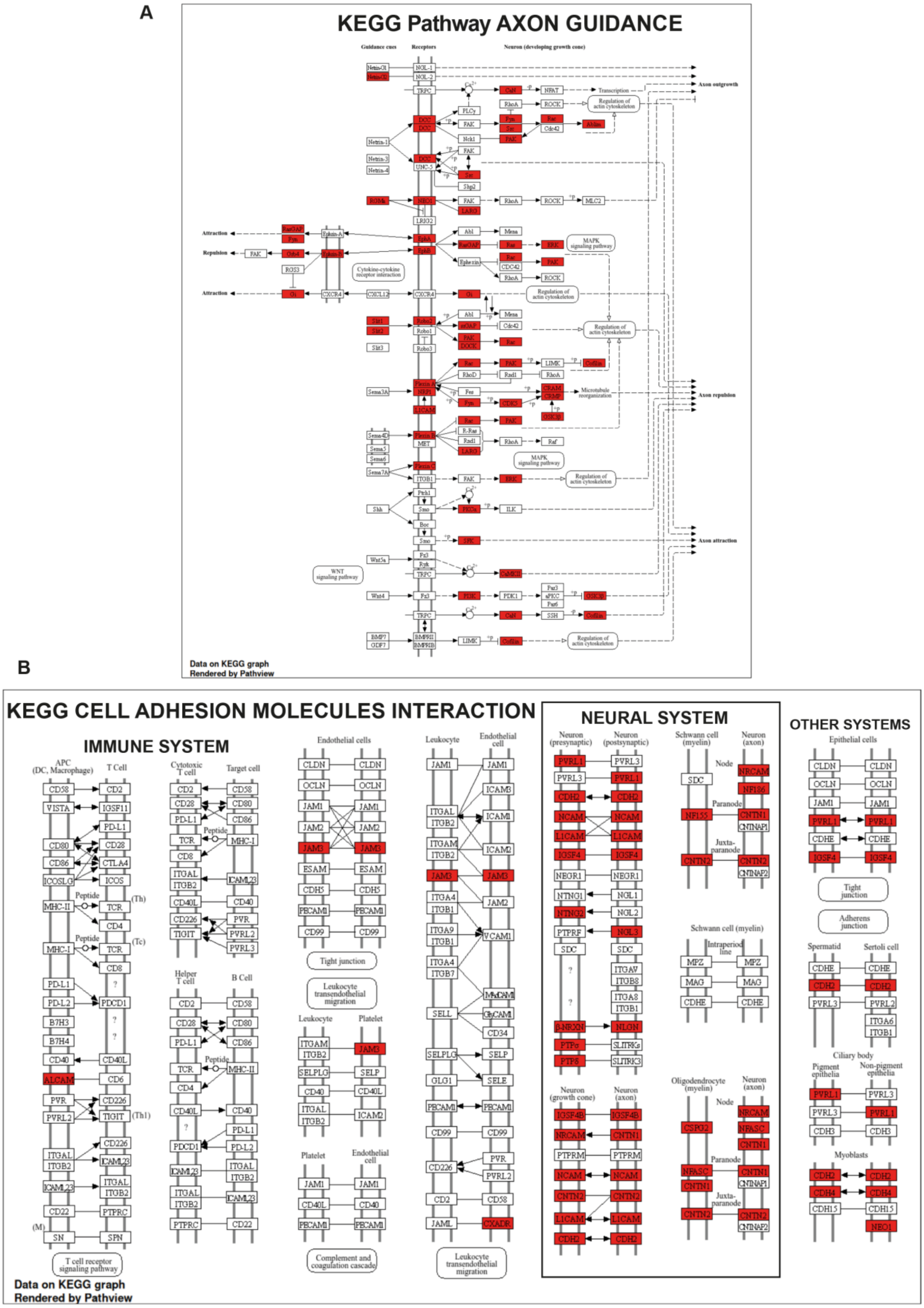

Figure S7. The KEGG pathways for axon guidance and cell adhesion molecule interactions are enriched in brain organoids

Gene Ontology (GO) was performed on the list of differentially expressed proteins between D21

organoids and ESCs using ShinyGO. This led to the identification of the KEGG pathways for ‘axon guidance’ (A) and ‘cell adhesion molecules interaction’ (B). The retrieved proteins are in red.

A

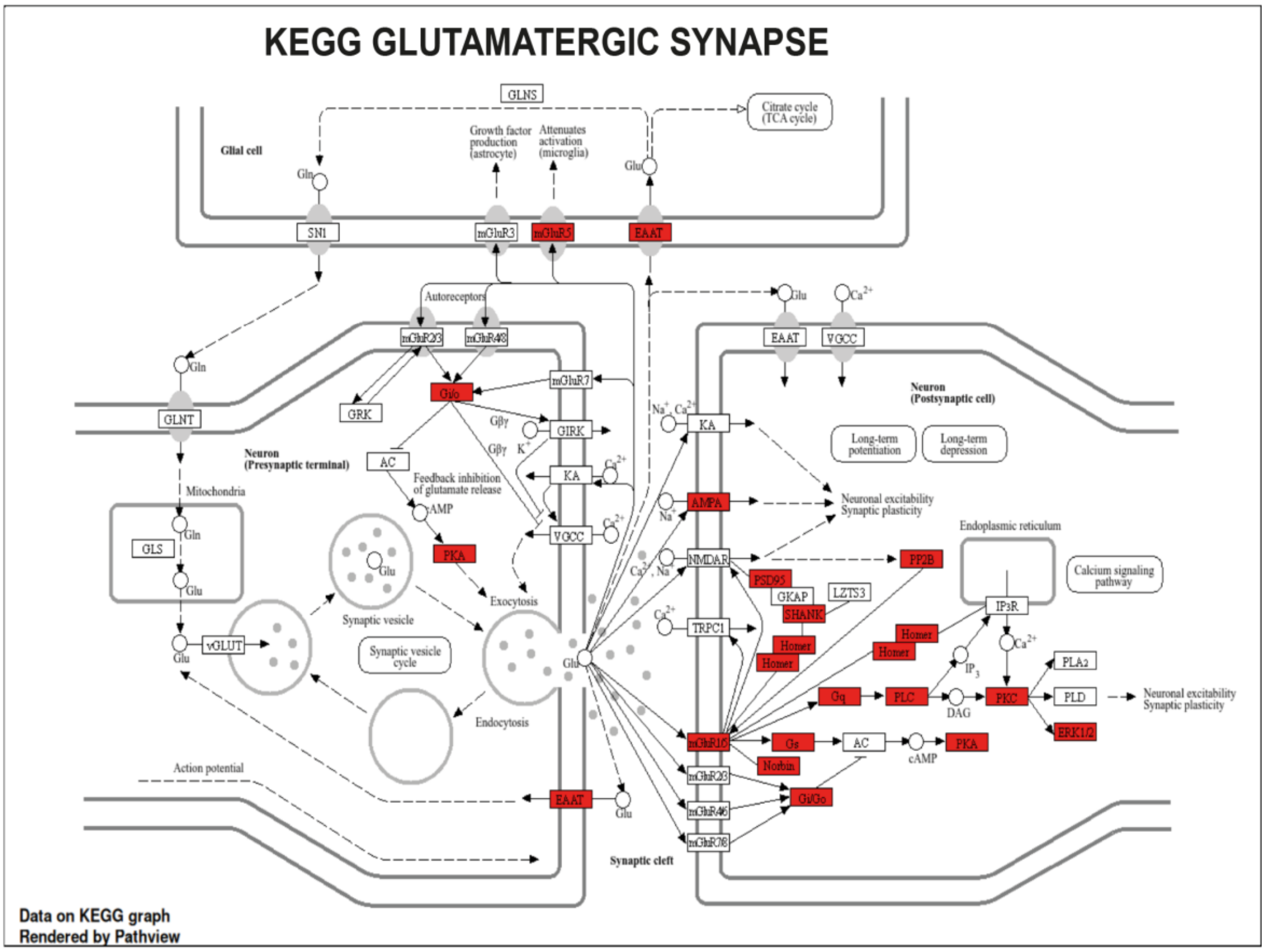

KEGG GLUTAMATERGIC SYNAPSE
GLNS
Citrate cycle (TCA cycle)
Glial cell
Growth factor production (astrocyte)
Attenuates activation (microglia)
Gln
Glu
SN1
mGluR3
mGluR5
EAAT
Autoreceptors
mGluR2/3
mGluR4/8
Gln
GLNT
Gi/o
GRK
mGluR7
Gβγ
GIRK
K+
AC
Feedback inhibition of glutamate release
KA
cAMP
PKA
VGCC
Neuron (Presynaptic terminal)
Mitochondria
Gln
GLS
Glu
Synaptic vesicle
vGLUT
Glu
Exocytosis
Synaptic vesicle cycle
Endocytosis
Action potential
Glu
Synaptic cleft
Na+
Ca2+
EAAT
VGCC
Neuron (Postsynaptic cell)
KA
Long-term potentiation
Long-term depression
AMPA
Neuronal excitability Synaptic plasticity
NMDAR
PP2B
Endoplasmic reticulum
PSD95
GKAP
LZTS3
SHANK
Calcium signaling pathway
IP3R
TRPC1
Homer
Homer
Homer
Homer
IP3
Gq
PLC
DAG
PKC
PLA2
PLD
Neuronal excitability Synaptic plasticity
ERK1/2
mGluR1/5
Gs
AC
cAMP
PKA
Norbin
mGluR2/3
Gi/Go
mGluR4/6
mGluR7/8
Data on KEGG graph
Rendered by Pathview

B

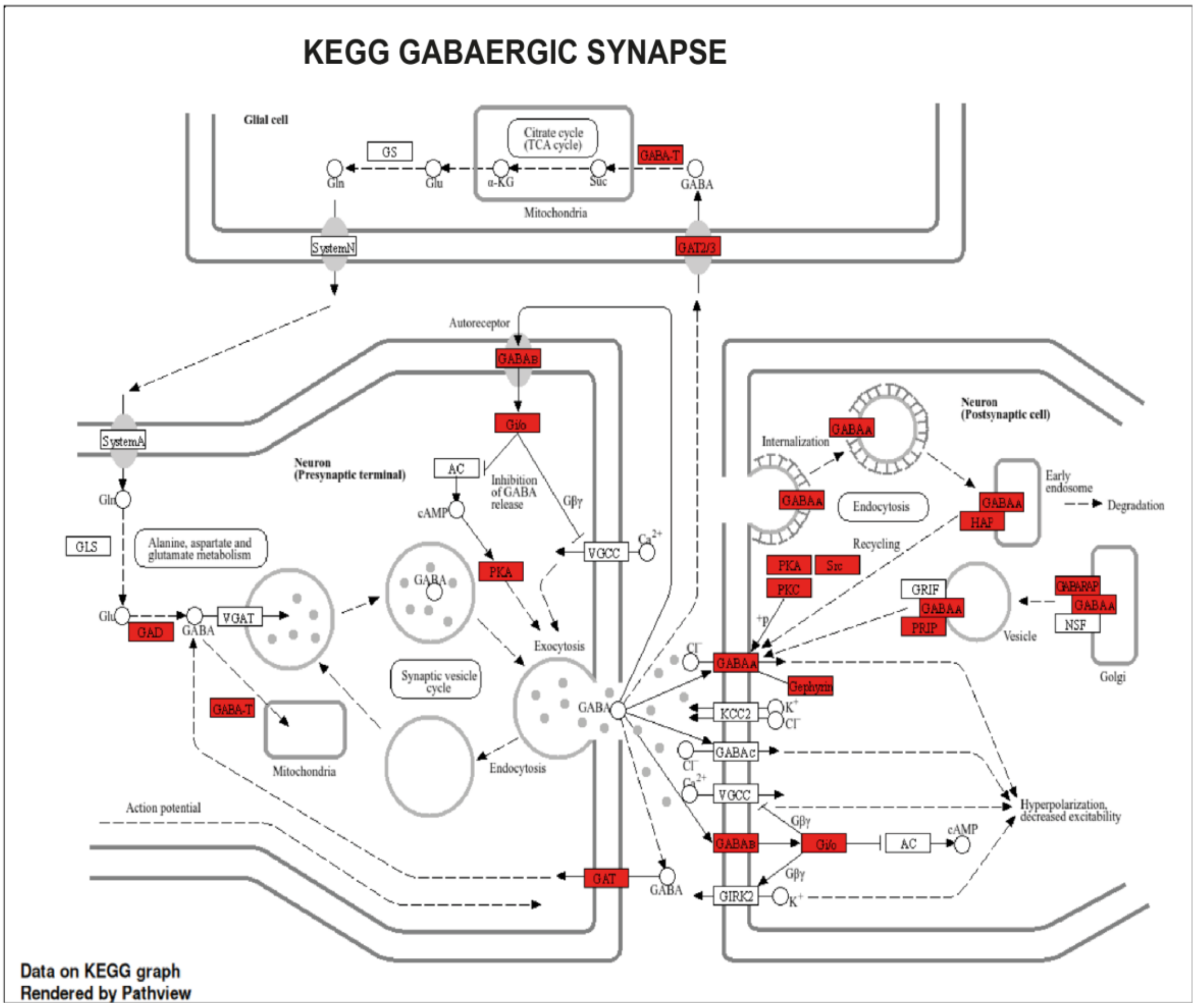

KEGG GABAERGIC SYNAPSE
Glial cell
Citrate cycle (TCA cycle)
GS
GABA-T
Gln
Glu
α-KG
Suc
GABA
Mitochondria
SystemN
GAT2/3
Autoreceptor
GABAB
Gi/o
SystemA
Neuron (Presynaptic terminal)
AC
Inhibition of GABA release
cAMP
Gβγ
Gln
GLS
Alanine, aspartate and glutamate metabolism
VGCC
Ca2+
PKA
GABA
Glu
GAD
GABA
VGAT
Exocytosis
Synaptic vesicle cycle
GABA-T
Mitochondria
Endocytosis
GABA
Action potential
GAT
GABA
Neuron (Postsynaptic cell)
GABAA
Internalization
GABAA
Endocytosis
GABAA
HAP
Early endosome
Degradation
Recycling
PKA
Src
PKC
GRIF
GABAA
PRIP
GABARAP
GABAA
NSF
Vesicle
Golgi
Cl-
GABAA
Gephyrin
KCC2
K+
Cl-
GABAC
Cl-
Ca2+
VGCC
Gβγ
GABAB
Gi/o
AC
cAMP
Hyperpolarization, decreased excitability
Gβγ
GIRK2
K+
Data on KEGG graph
Rendered by Pathview

Figure S8. The KEGG pathways for glutamatergic and GABAergic synapses are enriched in brain organoids

Gene Ontology (GO) was performed on the list of differentially expressed proteins between D21 organoids and ESCs using ShinyGO. This led to the identification of the KEGG pathways for 'glutamatergic synapse' (A) and 'GABAergic synapse' (B). The retrieved proteins are in red.

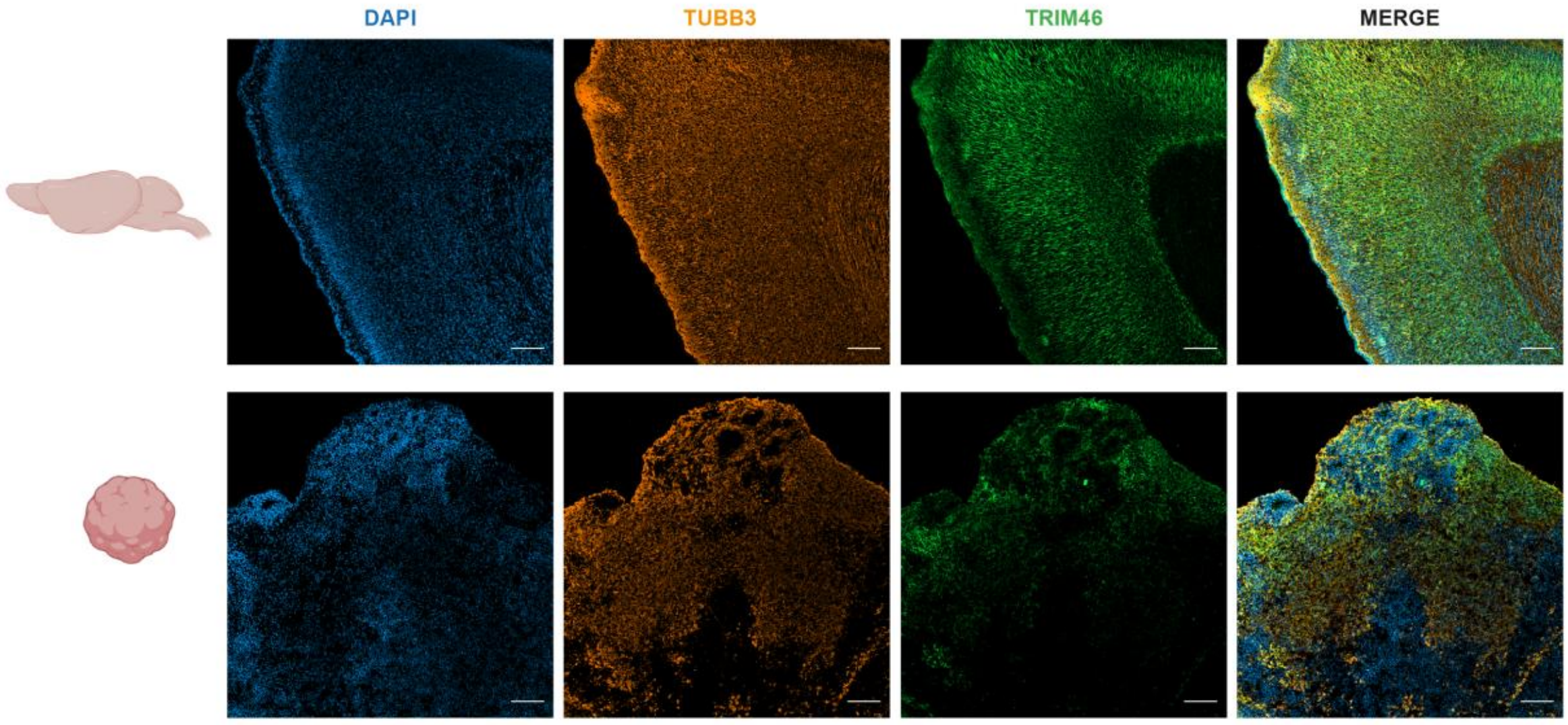

Figure S9. Brain organoids do not reproduce the parallel organisation of newly specified axons
Immunostaining of newly specified axons, labelled with an antibody to TRIM46 (green) and neurons with an antibody to TUBB3 (TUJ1, orange) in P1 mouse brain (top) and in D21 organoids (bottom). Nuclei were stained with DAPI. Scale bars: 100 µm.

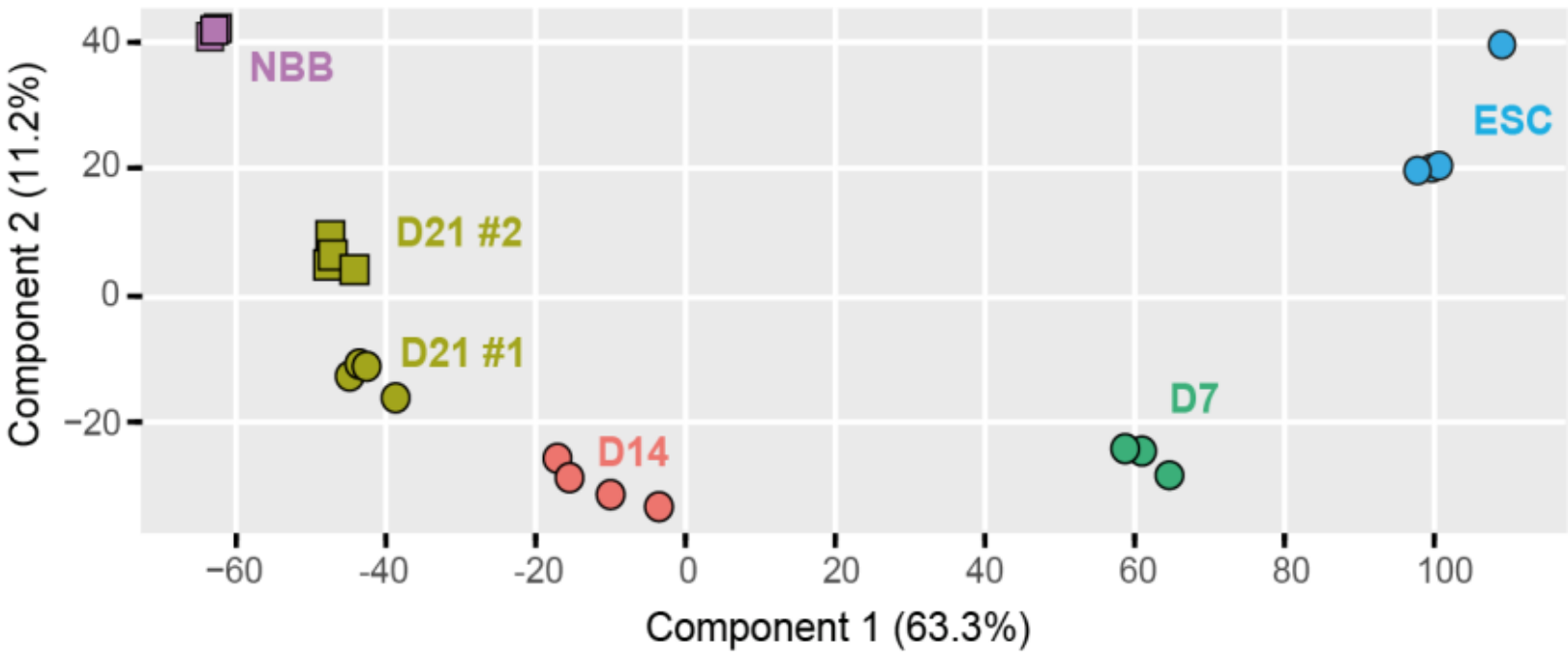

Figure S10. The proteome of brain organoids progressively approaches that of the NBB
PCA showing the distribution of the proteomes of mESCs, brain organoids (at days 7, 14, and 21), and NBB samples. Note the proximity between D21 samples from the two rounds of proteomics analysis.

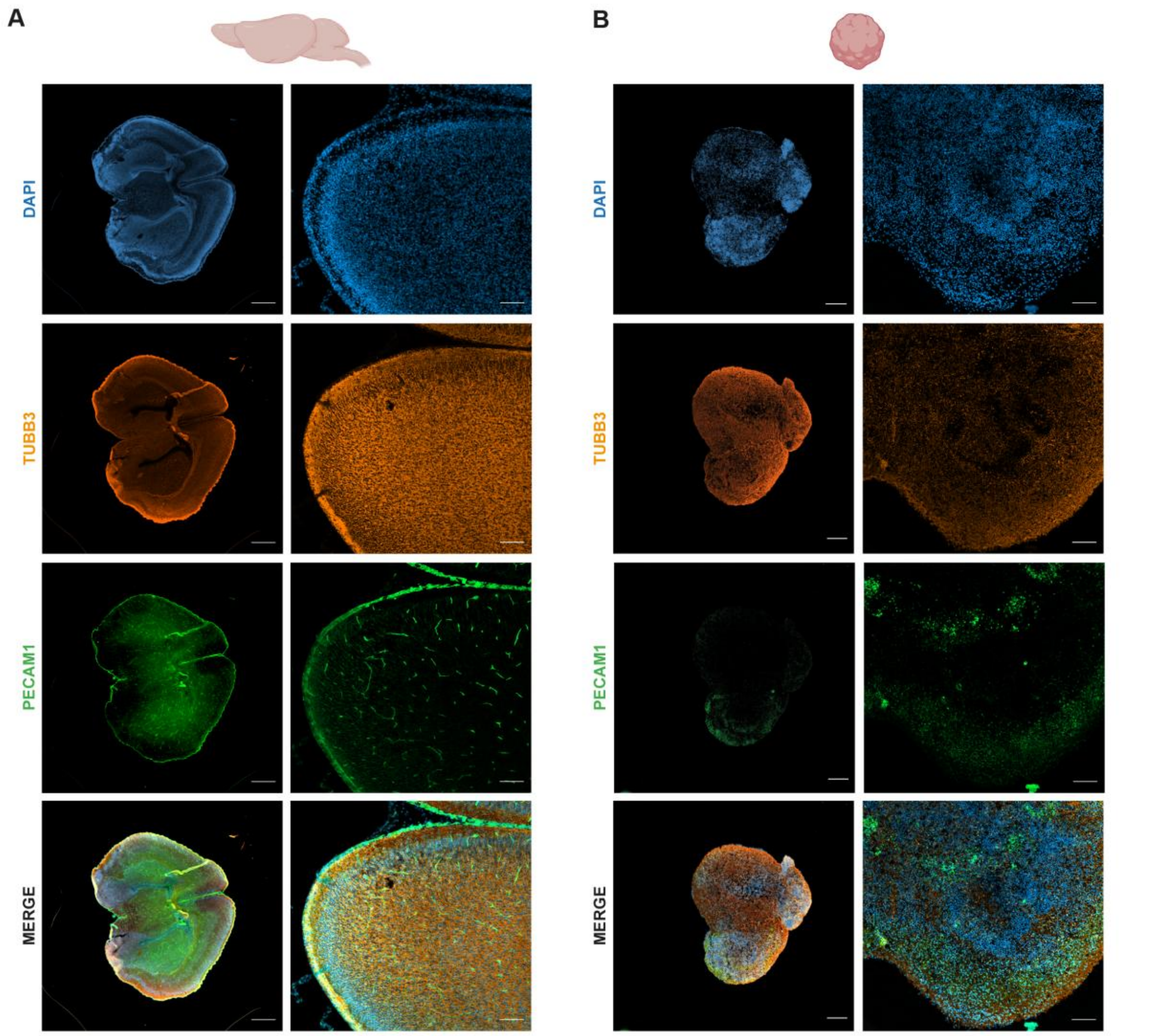

Figure S11. Absence of blood vessels in brain organoids
(Extended version of Figure 7D)
(A) Immunostaining of PECAM1/CD31 (green) and neurons with an antibody to TUBB3 (orange) in P1 mouse brain. Scale bars: 500µm (left); 100 µm (right). Part of the figure was created with BioRender.
(B) Same as in A in D21 organoids (bottom). Scale bars: 200µm (left), 100µm (right).

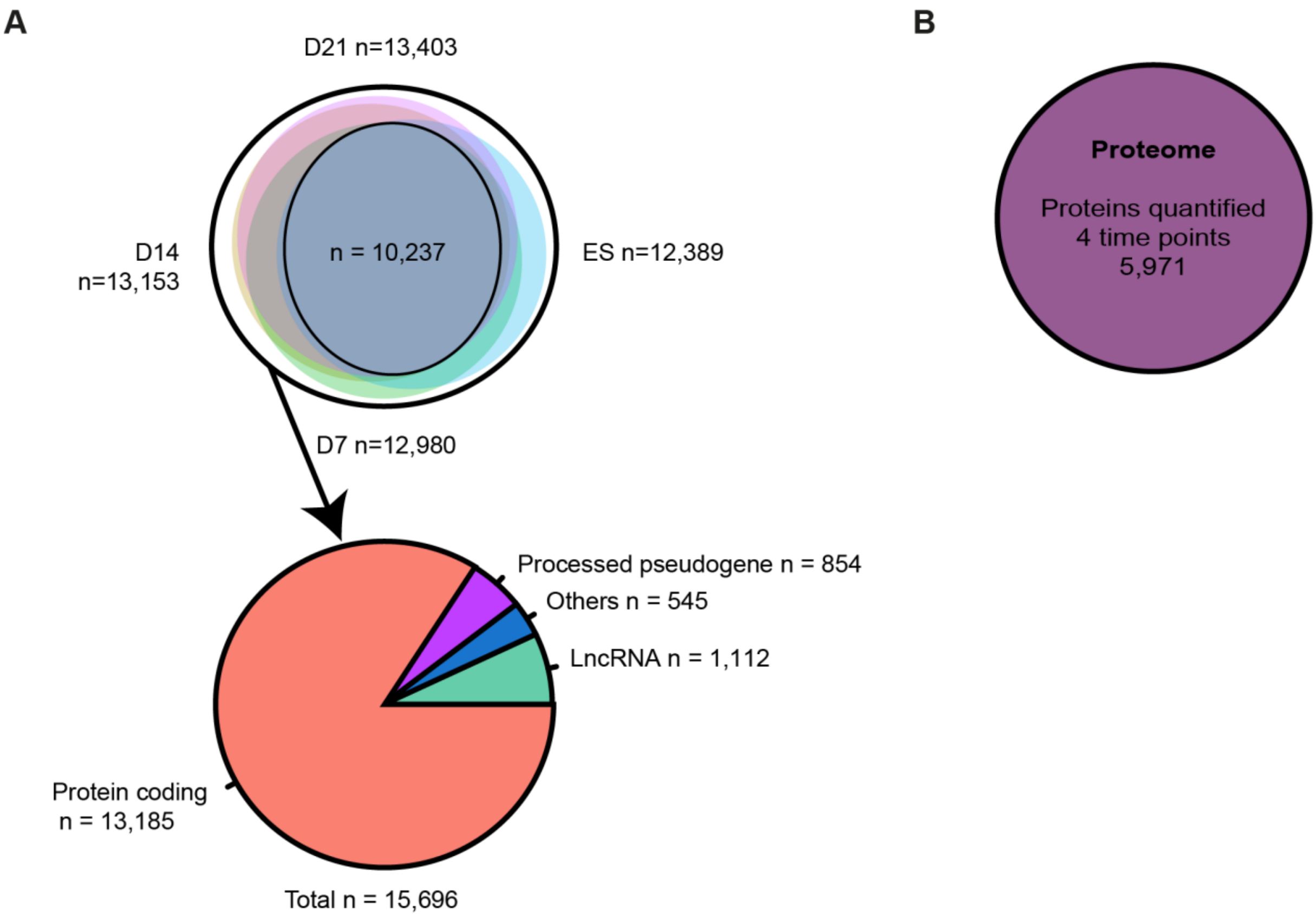

Figure S12. Number of quantified genes and proteins
(A) Gene annotation of the 15696 quantified genes with an average expression >1 FPKM in at least one group of samples.
(B) Output of proteomics: 5971 proteins with at least 3 peptides were quantified in all replicates of at least one condition.

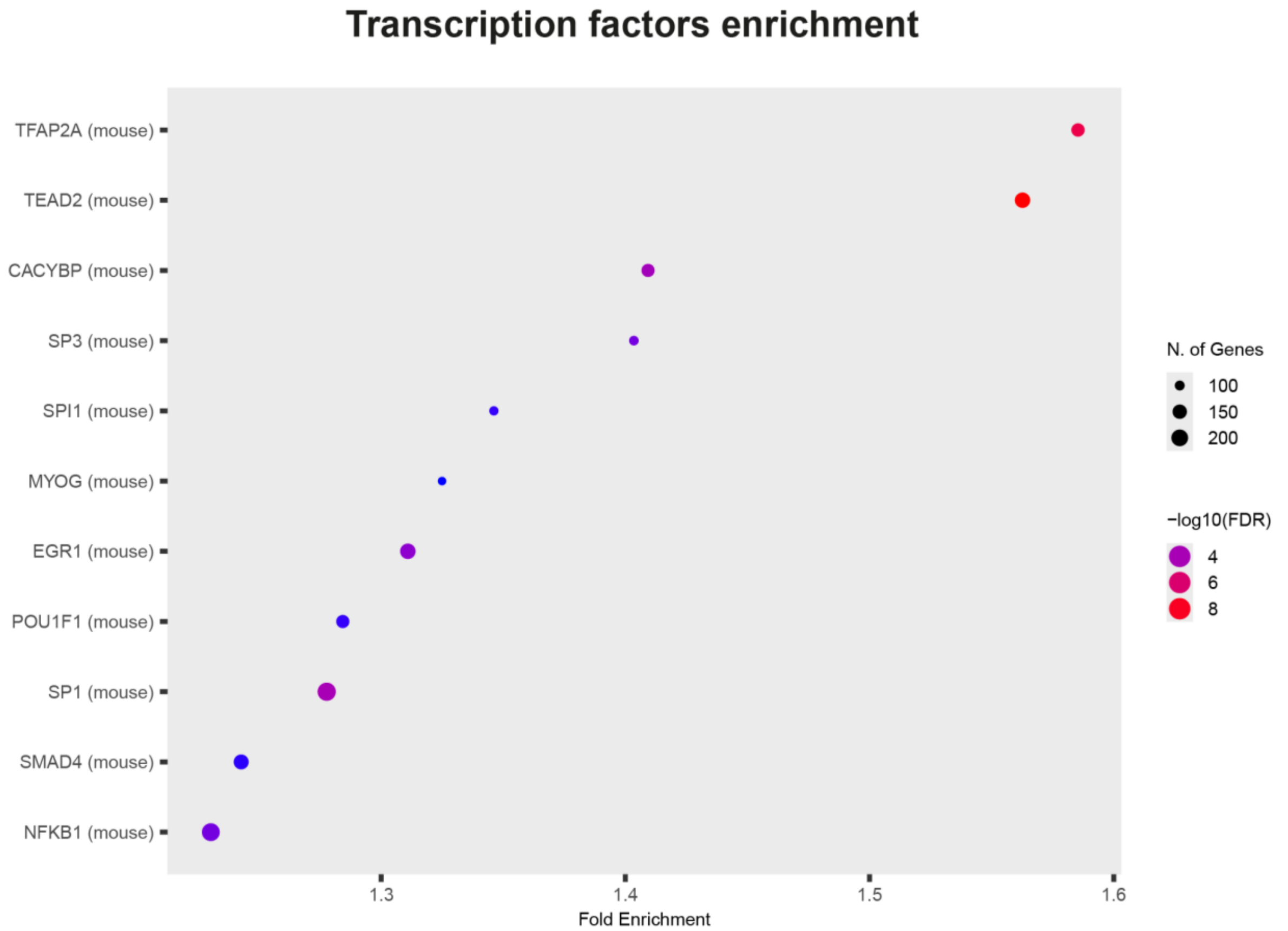

Figure S13. Transcription factors regulating protein networks during brain organoid formation
Transcription factors were identified according to their known targets among the list of the upregulated DEPs in D21 brain organoids compared to mESCs using TF.Target.TRANSFAC.and.JASPAR.

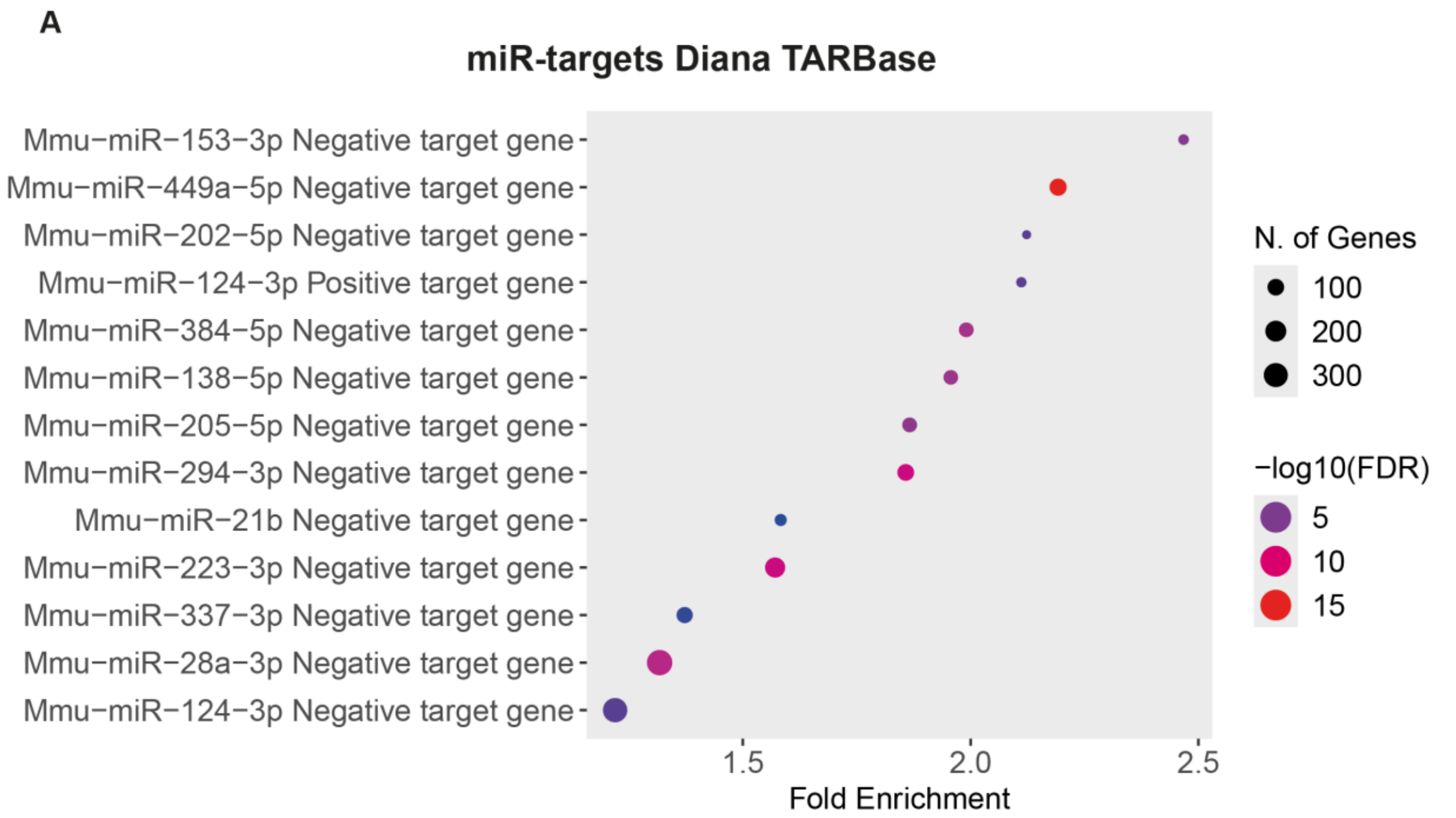

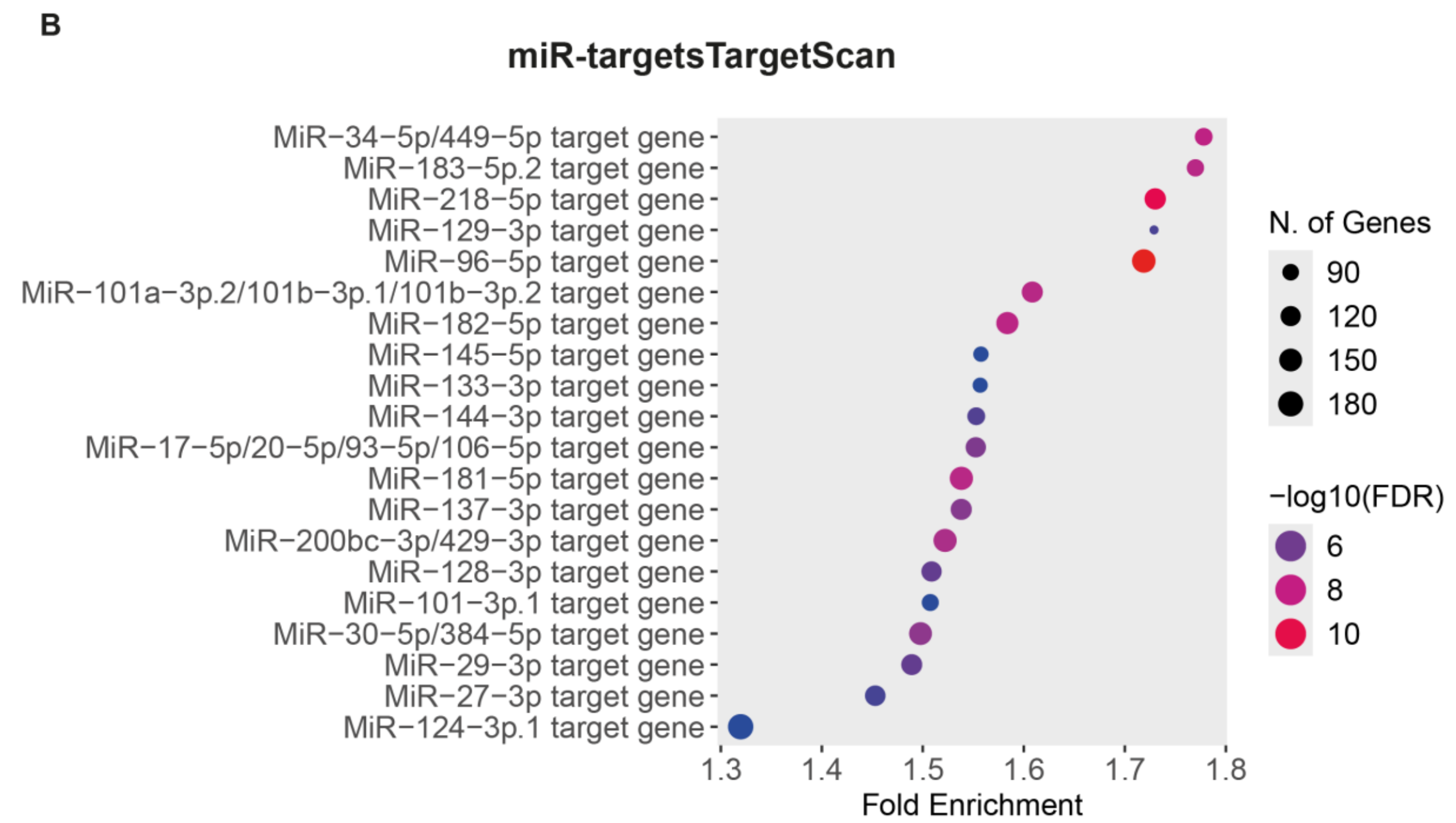

Figure S14. MicroRNAs regulating protein networks during brain organoid formation
MicroRNAs were identified according to their known targets among the list of the upregulated DEPs in D21 brain organoids compared to mESCs using miR-targets Diana TARBase (A) and miR-targetsTargetScan (B).

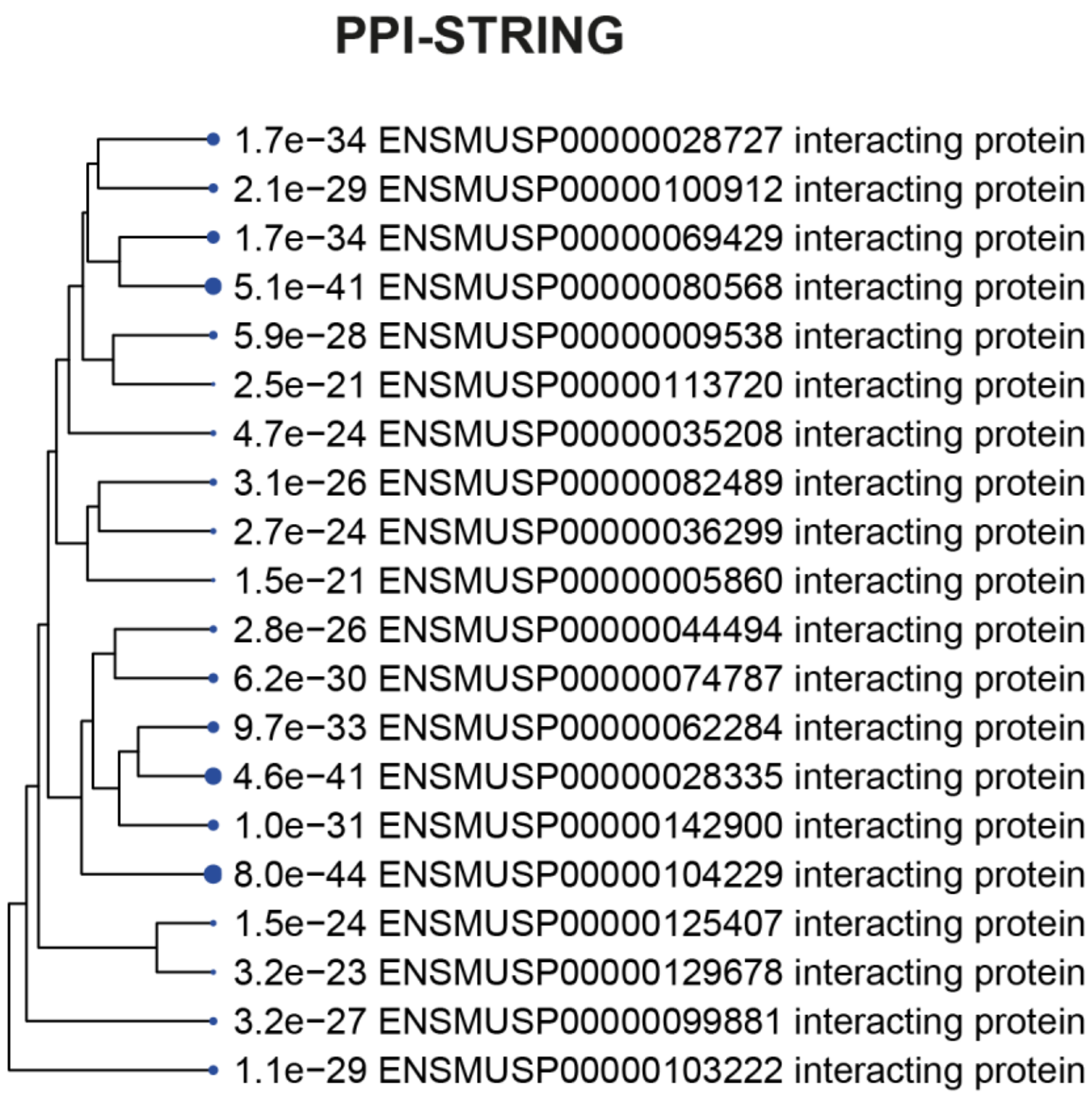

Figure S15. Protein-protein interaction networks during brain organoid formation
The list of upregulated DEPs in D21 brain organoids compared to mESCs was analysed for PPI using STRING.
